# Immune checkpoint inhibitor‐associated pituitary‐adrenal dysfunction: A systematic review and meta‐analysis

**DOI:** 10.1002/cam4.2661

**Published:** 2019-11-03

**Authors:** Jingli Lu, Lulu Li, Yan Lan, Yan Liang, Haiyang Meng

**Affiliations:** ^1^ Department of Pharmacy The First Affiliated Hospital of Zhengzhou University Zhengzhou Henan China; ^2^ Henan Key Laboratory of Precision Clinical Pharmacy Zhengzhou University Zhengzhou Henan China; ^3^ Department of Pharmacy Wuhan No.1 Hospital Wuhan Hubei China; ^4^ Department of Pharmacy Huangshi Center Hospital Huangshi Hubei China

**Keywords:** adrenal insufficiency, hypophysitis, hypopituitarism, immune checkpoint inhibitors

## Abstract

With the growing use of immune checkpoint inhibitors (ICIs), case reports of rare yet life‐threatening pituitary‐adrenal dysfunctions, particularly for hypopituitarism, are increasingly being published. In this analysis, we focus on these events by including the most recent publications and reports from early phase I/II and phase III clinical trials and comparing the incidence and risks across different ICI regimens. PubMed, Embase, and the Cochrane Library were systematically searched from inception to April 2019 for clinical trials that reported on pituitary‐adrenal dysfunction. The rates of events, odds ratios (ORs), and 95% confidence intervals (CIs) were obtained using random effects meta‐analysis. The analyses included data from 160 trials involving 40 432 participants. The rate was 2.43% (95% CI, 1.73%‐3.22%) for all‐grade adrenal insufficiency and 3.25% (95% CI, 2.15%‐4.51%) for hypophysitis. Compared with the placebo or other therapeutic regimens, ICI agents were associated with a higher incidence of serious‐grade adrenal insufficiency (OR 3.19, 95% CI, 1.84 to 5.54) and hypophysitis (OR 4.77, 95% CI, 2.60 to 8.78). Among 71 serious‐grade hypopituitarism instances in 12 336 patients, there was a significant association between ICIs and hypopituitarism (OR 3.62, 95% CI, 1.86 to 7.03). Substantial heterogeneity was noted across the studies for the rates of these events, which in part was attributable to the different types of ICIs and varied phases of the clinical trials. Although the rates of these events were low, the risk was increased following ICI‐based treatment, particularly for CTLA‐4 inhibitors, which were associated with a higher incidence of pituitary‐adrenal dysfunction than PD‐1/PD‐L1 inhibitors.

## Graphical abstract

Although the rates were rare, immune checkpoint inhibitor‐based treatment was associated with a higher incidence of pituitary‐adrenal dysfunction, particularly for CTLA‐4 inhibitors.

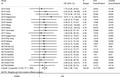

## 1 INTRODUCTION

Immune checkpoint inhibitors (ICIs), including anti‐cytotoxic T‐lymphocyte antigen‐4 (CTLA‐4), anti‐programmed cell death 1 (PD‐1), and anti‐programmed death 1 ligand 1 (PD‐L1), have become a mainstay of treatment for many types of cancers.^1^ As a consequence of the favorable response rates and the improved survival provided by ICIs, these agents continue to undergo extensive evaluation for the treatment of additional tumor types, thus expanding the number of patients exposed to ICIs.^2^

Antitumor immunotherapies using ICIs target T‐cell‐negative feedback loops, augmenting the immune response to attack cancer cells.^3^ However, unwanted consequences of their mechanisms of action lead to a unique spectrum of adverse events, most of which are immune‐related adverse events (irAEs).^4^ irAEs can be observed in most organs, with varying frequencies and severities.^5^ Endocrine dysfunction following the use of ICIs has emerged as one of the most common irAEs.^6^, ^7^ Rare yet life‐threatening pituitary‐adrenal dysfunctions, including hypophysitis, adrenal insufficiency and hypopituitarism, have been constantly reported recently, which raises new concerns around ICIs.^8^ Although a previous meta‐analysis has been conducted,^9^, ^10^, ^11^ none of these studies reported ICI‐associated hypopituitarism, which is rarely reversible and often requires prolonged or life‐long substitutive hormonal treatment. Given the emergence of substantive new data, we update this review and focus on pituitary‐adrenal dysfunction by including the most recent publications and reports from clinical trials, comparing the incidence and risks of these adverse events across different ICI regimens.

## 2 METHODS AND MATERIALS

### 2.1 Search methods and study selection

This systematic review and meta‐analysis was performed in adherence with the Preferred Reporting Items for Systematic Reviews and Meta‐Analyses (PRISMA) guidelines.^12^ Scientific literature searches were conducted in the PubMed, Embase, and Cochrane Library databases (Cochrane Central Register of Controlled Trials) from the inception of all searched databases to April 2019. We also searched the https://ClinicalTrials.gov website to identify completed but not yet published reports. Relevant text words that consisted of terms including ‘phase’ and the following terms were used: ipilimumab, MDX‐010, tremelimumab, CP‐675206, nivolumab, BMS‐963558, pembrolizumab, MK‐3475, atezolizumab, MPDL3280A, avelumab, MSB0010718C, durvalumab, MEDI4736, cemiplimab, REGN2810, toripalimab, JS001, sintilimab, and IBI308 (Table S1). Because the aim of this study was to assess the risk of ICI‐associated pituitary‐adrenal adverse events, only studies in the English language that reported adrenal sufficiency, hypophysitis, or hypopituitarism events in adult participants receiving ICIs were included. Two authors screened potentially eligible scientific reports for full‐text review (LL and YL). Three authors (JL, YL and HM) reviewed and selected the full‐text articles for data extraction. Any disagreements were resolved by consensus.

### 2.2 Data extraction

For the included studies, data were extracted independently by two of the three authors (JL, LL and YL). Any discrepancies in data extraction were resolved by consensus. The retrieved data included author name, year of publication, registry number of trials, phase, cancer type, type of ICIs, number of patients, and number of patients with all‐grade and serious‐grade pituitary‐adrenal dysfunction (adrenal sufficiency, hypophysitis or hypopituitarism events). Because adverse events were categorized into serious or other in reports from https://ClinicalTrials.gov, we identified grades 3‐5 as serious for data from published reports based on the Common Terminology Criteria for Adverse Events (CTCAE) categorization. If the number of pituitary‐adrenal associated adverse events was not reported in the published reports, but the corresponding registry report from the https://ClinicalTrials.gov website was reported, we used the safety outcome data from the https://ClinicalTrials.gov website. If data were reported from both sources, we used data from reports where the data were more complete. If multiple publications reported the same trial, only the most relevant and complete publications were used.

### 2.3 Statistical analysis

For event rates, the data were transformed using the Freeman‐Tukey double arcsine transformation and pooled using a random effects model. We explored the sources of heterogeneity based on subgroup analysis: type of ICIs (PD‐1 inhibitors vs PD‐L1 inhibitors vs CTLA‐4 inhibitors vs combination of ICIs) and phase (phase 1 vs phase 2 vs phase 3). The statistical analyses of event rates were performed using R statistical software (meta package, R Foundation). For the randomized controlled clinical trials, the odds ratio (OR) of pituitary‐adrenal dysfunction and their associated 95% confidence intervals (CIs) were calculated in the ICI group compared with the control group. The individual study ORs were pooled using Peto's method because of the low rates of adverse events. The statistical analyses of ORs were performed using STATA (version 15).

The heterogeneity across the trials for each outcome was estimated using the *I* ^2^ statistic and by calculating the *P* value. An *I* ^2^ statistic of 0%‐25%, 26%‐75% and 76%‐100% was considered to reflect low, moderate, and high heterogeneity, respectively. A *P* value of less than .05 was defined as significant heterogeneity. Publication bias and small study effects were assessed using Egger's test and the Begg correlation test, and a *P* value less than .1 was defined as significant publication bias.

## 3 RESULTS

### 3.1 Eligible studies and characteristics

**Figure 1:**
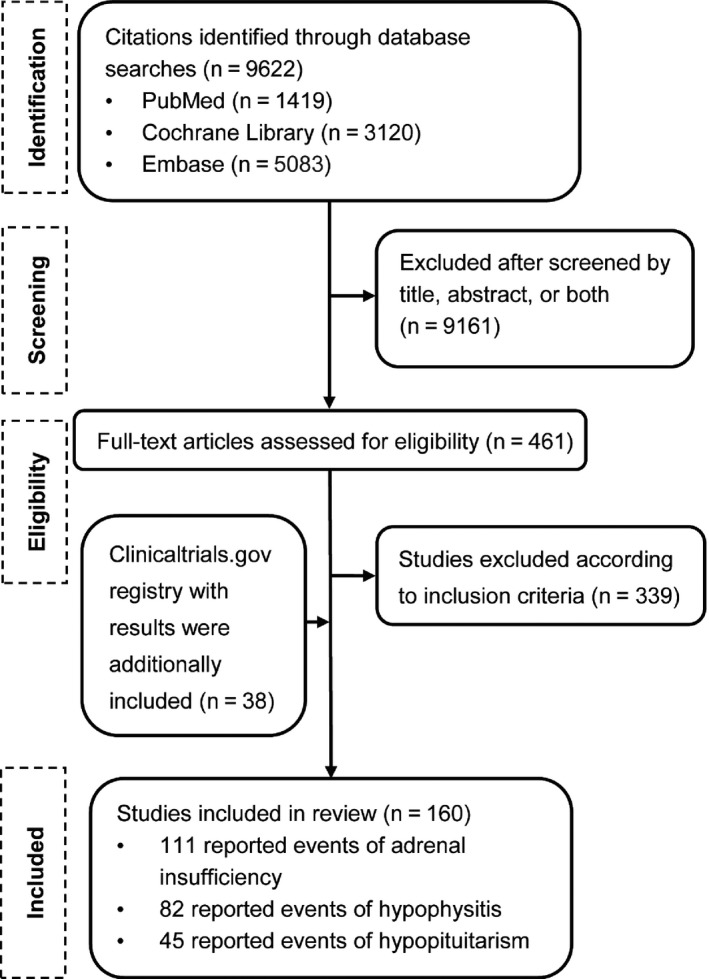
Flow diagram of the literature search

The search of literature and review of references yielded 9622 potentially eligible studies. After excluding duplicates and references that did not describe clinical trials assessing ICIs for cancers, 461 references were retrieved for further assessment. A total of 122 studies that fulfilled our inclusion criteria were included in the analyses. In addition, 38 clinical trials with results from https://ClinicalTrials.gov were identified and included. Overall, we included a total of 160 clinical trials involving 40 432 patients in the meta‐analysis (Figure 1, Table S2).^13^, ^14^, ^15^, ^16^, ^17^, ^18^, ^19^, ^20^, ^21^, ^22^, ^23^, ^24^, ^25^, ^26^, ^27^, ^28^, ^29^, ^30^, ^31^, ^32^, ^33^, ^34^, ^35^, ^36^, ^37^, ^38^, ^39^, ^40^, ^41^, ^42^, ^43^, ^44^, ^45^, ^46^, ^47^, ^48^, ^49^, ^50^, ^51^, ^52^, ^53^, ^54^, ^55^, ^56^, ^57^, ^58^, ^59^, ^60^, ^61^, ^62^, ^63^, ^64^, ^65^, ^66^, ^67^, ^68^, ^69^, ^70^, ^71^, ^72^, ^73^, ^74^, ^75^, ^76^, ^77^, ^78^, ^79^, ^80^, ^81^, ^82^, ^83^, ^84^, ^85^, ^86^, ^87^, ^88^, ^89^, ^90^, ^91^, ^92^, ^93^, ^94^, ^95^, ^96^, ^97^, ^98^, ^99^, ^100^, ^101^, ^102^, ^103^, ^104^, ^105^, ^106^, ^107^, ^108^, ^109^, ^110^, ^111^, ^112^, ^113^, ^114^, ^115^, ^116^, ^117^, ^118^, ^119^, ^120^, ^121^, ^122^, ^123^, ^124^, ^125^, ^126^, ^127^, ^128^, ^129^, ^130^, ^131^, ^132^, ^133^, ^134^, ^135^, ^136^, ^137^, ^138^, ^139^, ^140^, ^141^, ^142^, ^143^, ^144^, ^145^, ^146^, ^147^, ^148^, ^149^, ^150^, ^151^, ^152^, ^153^, ^154^, ^155^, ^156^, ^157^, ^158^, ^159^, ^160^, ^161^, ^162^, ^163^, ^164^, ^165^, ^166^, ^167^, ^168^, ^169^, ^170^, ^171^, ^172^ The trials include 37 phase 3 studies with 25 084 patients; 1 phase 2/3 study with 1033 patients; 66 phase 2 studies with 8529 patients; 11 phase 1/2 studies with 1263 patients; and 45 phase 1 studies with 4523 patients. The ICIs used included PD‐1 inhibitors (n = 88 cohorts; n = 13 519 patients), PD‐L1 inhibitors (n = 29 cohorts; n = 4532 patients), CTLA‐4 inhibitors (n = 102 cohorts; n = 9000 patients), and combination with PD‐1/PD‐L1 plus CTLA‐4 inhibitors (n = 37 cohorts; n = 2952 patients). The most common disease types were melanoma (n = 60 studies; n = 14 073 patients) and non‐small‐cell lung cancer (n = 29 studies; n = 12 082 patients) (Table 1).

**Table 1:** Study and patient characteristics

Study Characteristic	Studies, No.	Patients, No
Total	160	40 432
Phase		
1	45	4523
1/2	11	1263
2	66	8529
2/3	1	1033
3	37	25 084
ICI type (cohort)		
PD‐1 inhibitors	88	13 519
PD‐L1 inhibitors	29	4532
CTLA‐4 inhibitors	102	9000
Combination	37	2952
Common cancer type		
Melanoma	60	14 073
Non‐small‐cell lung cancer	29	12 082
Sponsorship		
Pharmaceutical companies	139	39 274
Others	21	1158
Reporting year		
2015 or before	48	10 308
2016	17	5008
2017	28	8014
2018	45	10 416
2019 (up to May)	22	6686

### 3.2 Rates of adrenal insufficiency

The rate of all‐grade adrenal insufficiency ranged from 0% to 64%, and the rate of serious‐grade adrenal insufficiency ranged from 0% to 33.3%. One study did not report the number of events^125^; across the other studies, 289 cases of any‐grade adrenal insufficiency were observed among 12 295 patients, and 176 cases of serious‐grade adrenal insufficiency were observed among 22 103 patients. Using a random effects model, the rates of all‐grade and serious‐grade adrenal insufficiency were 2.43% (95% CI, 1.73%‐3.22%) and 0.15% (95% CI, 0.05%‐0.29%), respectively (Table 2). There was some evidence of heterogeneity as quantified by *I* ^2^ statistics of 73.6% and 42.3% for all‐grade and serious‐grade adrenal insufficiency, respectively (Table 2). In this analysis, publication bias was evident (Table S3).

**Table 2:** Incidence of immune checkpoint inhibitor‐associated pituitary‐adrenal dysfunction

Type	All‐grade adrenal insufficiency				Serious‐grade adrenal insufficiency				All‐grade hypophysitis				Serious‐grade hypophysitis			
	N	E/n	Rate	I^2^%	N	E/n	Rate	I^2^%	N	E/n	Rate	I^2^%	N	E/n	Rate	I^2^%
Overall	118	289/12 295	2.43 (1.73‐3.22)	73.6	159	176/22 103	0.15 (0.05‐0.29)	42.3	97	484/11 893	3.25 (2.15‐4.51)	87.0	126	240/17 389	0.44 (0.21‐0.74)	59.3
PD‐1 inhibitors	32	50/5113	0.49 (0.16‐0.93)	9.1	54	44/9296	0.03 (0.00‐0.11)	2.1	24	48/5983	0.41 (0.22‐0.66)	5.3	39	33/8271	0.06 (0.00‐0.17)	0.0
PD‐L1 inhibitors	15	18/1826	0.43 (0.02‐1.20)	34.6	26	17/4280	0.01 (0.00‐0.16)	13.6	1	1/88	/	/	0	0	/	/
CTLA‐4 inhibitors	48	136/3610	5.32 (3.30‐7.68)	79.4	53	71/6008	0.42 (0.07‐0.98)	56.5	54	304/4265	4.53 (2.62‐6.82)	83.5	64	155/6852	0.78 (0.29‐1.44)	60.1
Combination therapy	23	85/1746	4.05 (2.81‐5.45)	27.3	21	38/2182	0.89 (0.43‐1.47)	0.0	18	131/1557	7.68 (5.99‐9.54)	28.8	23	52/2266	1.66 (1.07‐2.34)	0.0
Phase 1	32	50/2071	2.25 (1.00‐3.85)	58.1	36	23/2317	0.25 (0.00‐0.88)	36.7	22	54/1439	4.53 (1.79‐8.11)	76.6	30	21/1937	0.31 (0.00‐1.03)	29.3
Phase 2 and 1/2	66	153/3968	3.89 (2.44‐5.58)	75.6	80	75/5982	0.48 (0.16‐0.92)	44.4	49	107/2521	3.40 (1.66‐5.55)	75.8	58	53/3660	0.47 (0.07‐1.12)	50.1
Phase 3 and 2/3	20	86/6256	1.30 (0.77‐1.94)	69.5	43	78/13 804	0.37 (0.23‐0.53)	28.5	26	323/7933	3.11 (1.62‐5.01)	94.3	38	166/11 792	1.03 (0.65‐1.49)	75.1

Note

Values for rates are percentages (95% confidence intervals).

Abbreviations: E, number of events; n, number of patients; N, number of cohorts.

Heterogeneity was explored by using subgroup analysis on the basis of the phase of the trial and type of ICIs. The results showed that anti‐CTLA‐4 was associated with higher rates of both high‐grade and serious‐grade adrenal insufficiency, with rates of 5.32% and 0.42%, respectively. Phase 2 trials showed a trend toward higher rates, with 3.89% for all‐grade adrenal insufficiency and 0.48% for serious‐grade adrenal insufficiency (Table 2).

**Figure 2:**
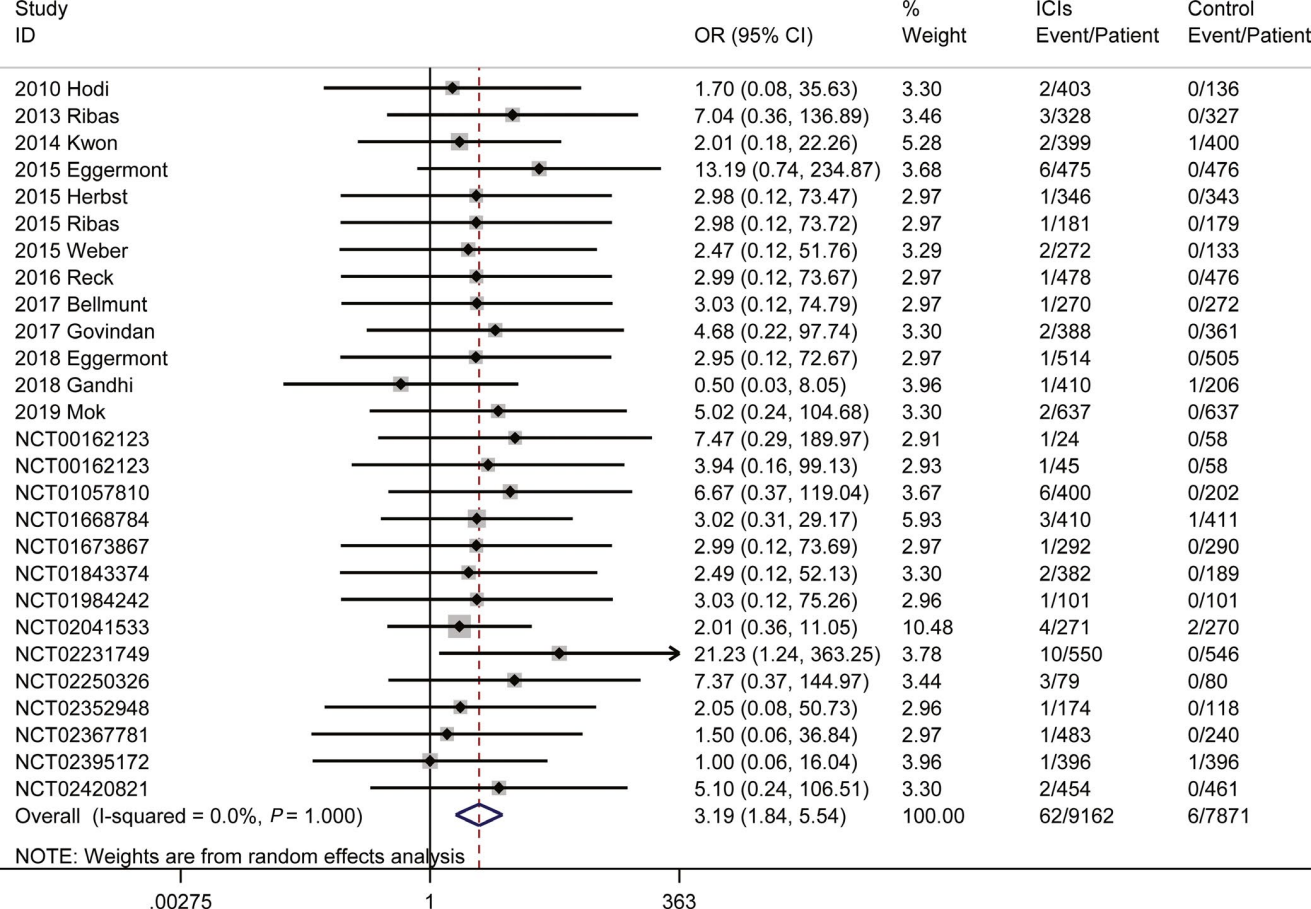
Risk of serious‐grade adrenal insufficiency in patients treated with ICIs vs controls

To assess the relative rate of ICI‐associated adrenal insufficiency compared with those in control arms, we calculated the relative risk of developing adrenal insufficiency in the randomized controlled clinical trials. Compared with patients in control arms, those treated with ICIs were at a higher risk for all‐grade (OR 2.63, 95% CI, 1.31%‐5.28%, Figure S1) and serious‐grade adrenal insufficiency (OR 3.19, 95% CI, 1.84%‐5.54%, Figure 2), with no significant between‐study heterogeneity. In this analysis, no publication bias was evident (Table S4).

### 3.3 Rates of hypophysitis

All‐grade hypophysitis was described in 61 studies and serious‐grade in 77 studies. Four studies did not report the number of events^41^, ^86^, ^110^, ^128^; across all the other studies, 484 cases of all‐grade hypophysitis in 11 893 patients and 240 cases of serious‐grade hypophysitis in 17 389 patients were reported. Pooling the data showed that the rates of all‐grade and serious‐grade hypophysitis were 3.25% (95% CI, 2.15%‐4.51%) and 0.44% (95% CI, 0.21%‐0.74%), respectively (Table 2). Substantial heterogeneity for all‐grade hypophysitis (I^2^ = 87.0%) and moderate heterogeneity for serious‐grade hypophysitis (I^2^ = 59.3%) were observed (Table 2). In this analysis, publication bias was evident (Table S3).

Subgroup analysis was performed to investigate heterogeneity based on the phase of the trial and type of ICIs. The rates of hypophysitis for all‐grade and serious‐grade patients were greatest with ICI combination therapy at 7.68% and 1.66%, respectively. For all‐grade hypophysitis, the rate was 4.53% with CTLA‐4 inhibitors and less than 1% with PD‐1 and PD‐L1 inhibitors. For serious‐grade hypophysitis, the rate was 0.78% for CTLA‐4 inhibitors and less than 0.1% for PD‐1 inhibitors. The phase of the trial was also associated with rates of hypophysitis. For all‐grade hypophysitis, the rate was 4.53% for phase 1 trials, 3.40% for phase 2 trials, and 3.11% for phase 3 trials. For serious‐grade hypophysitis, the rate was 0.31% for phase 1 trials, 0.47% for phase 2 trials, and 1.03% for phase 3 trials (Table 2).

**Figure 3:**
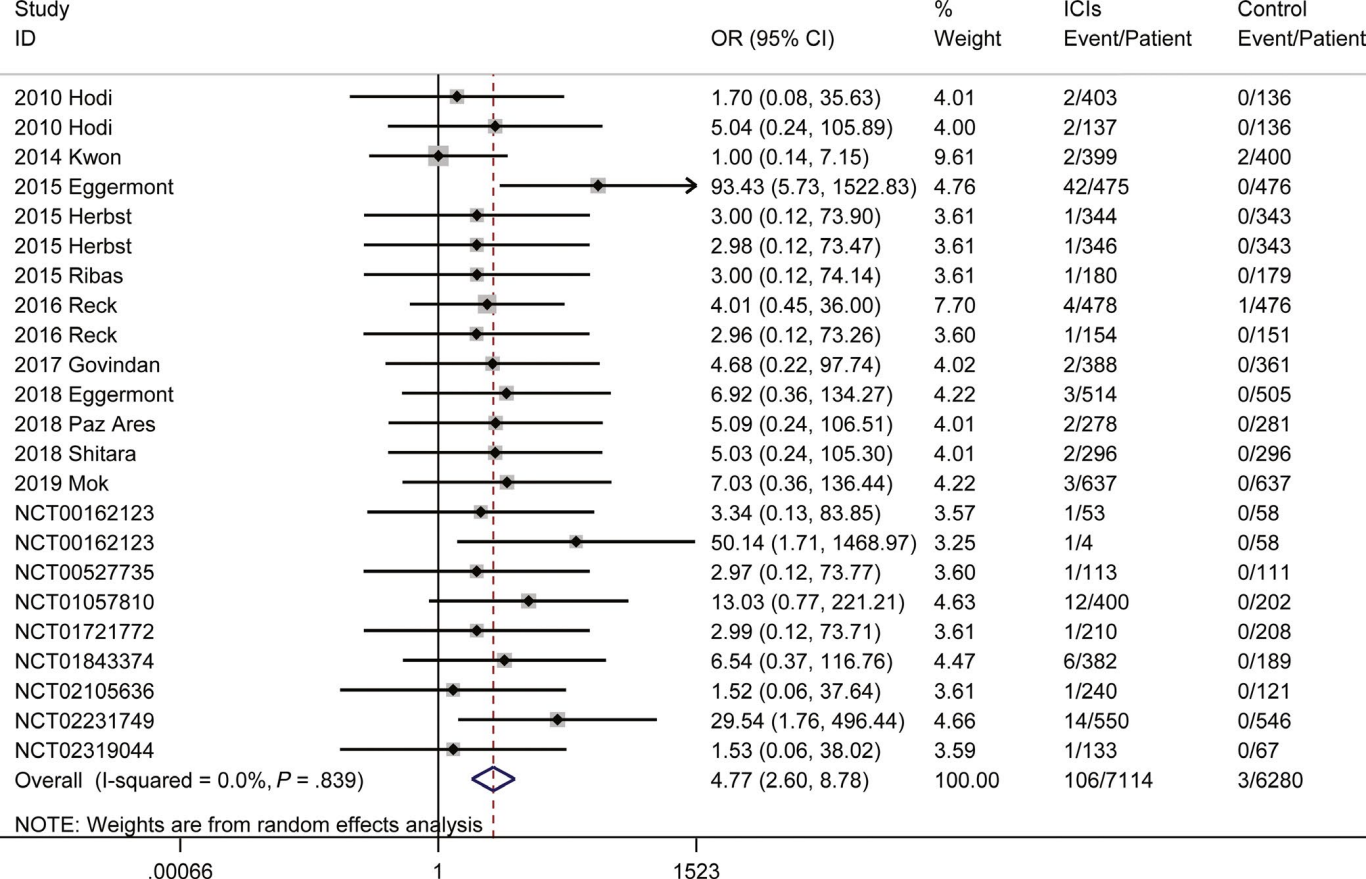
Risk of serious‐grade hypophysitis in patients treated with ICIs vs controls

Eleven clinical controlled trials (n = 7581 participants) assessed the relative risk of any‐grade hypophysitis, and 19 clinical controlled trials (n = 13 394 participants) assessed the relative risk of serious‐grade hypophysitis during treatment. Pooling the data of these studies showed that patients treated with ICIs were significantly more likely to experience all‐grade hypophysitis (OR 10.36, 95% CI, 5.08%‐21.12%, Figure S2) and serious‐grade hypophysitis (OR 4.77, 95% CI, 2.60%‐8.78%, Figure 3) than those treated with other regimens. There was no significant between‐study heterogeneity, with I^2^ statistics of 6.2% for all‐grade and 0% for serious‐grade hypophysitis. In this analysis, there was no significant publication bias (Table S4).

### 3.4 Rates of hypopituitarism

**Figure 4:**
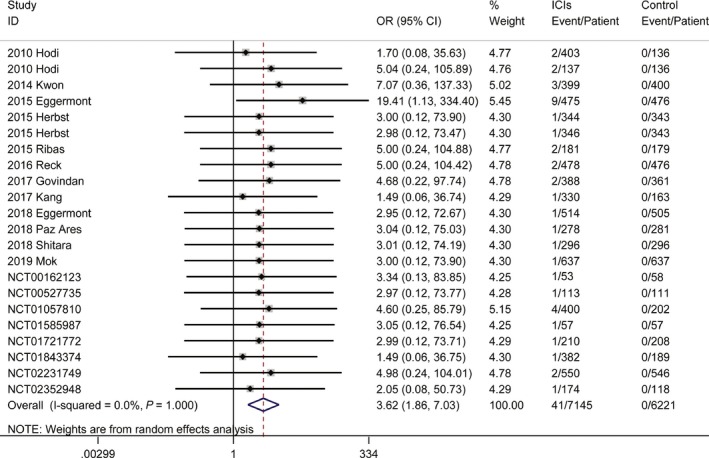
Risk of serious‐grade hypopituitarism in patients treated with ICIs vs controls

Regarding hypopituitarism, three studies did not report the number of events^33^, ^128^, ^173^; across the other studies, 45 cases of all‐grade hypopituitarism occurred in 3755 patients (raw event rate 1.20%), and 71 cases of serious‐grade hypopituitarism occurred in 12 336 patients (raw event rate 0.58%) who used at least one ICI. Due to the smaller number of events, no statistical inferences of the rates were made. For the randomized clinical controlled trials, the ORs of all‐grade and serious‐grade hypopituitarism in patients receiving ICIs compared with non‐ICI‐receiving control patients were 3.03 (95% CI, 0.52%‐17.57%, Figure S3) and 3.62 (95% CI, 1.86%‐7.03%, Figure 4), respectively. There was no substantial heterogeneity, with I^2^ statistics of 0% for both all‐grade and serious‐grade hypopituitarism. In this analysis, there was no significant publication bias (Table S4).

## 4 DISCUSSION

In this systematic review, we described ICI‐associated pituitary‐adrenal dysfunction in cancer patients. We extracted data from reports of academic publications and the https://ClinicalTrials.gov website to maximize the information gathered. Our study provides a systematic and quantitative analysis that assessed these effects and explored other important differences between trials regarding the rates of pituitary‐adrenal dysfunction in patients receiving ICIs.

We found five key points. First, from 160 clinical trials including 40 432 patients, we determined estimates of the rates of adrenal insufficiency and hypophysitis with ICIs of 2.43% and 3.25%, respectively. Second, the incidence of hypopituitarism was low, with raw event rates of 1.20% and 0.58% for all‐grade and serious‐grade hypopituitarism, respectively. Third, the rates of pituitary‐adrenal dysfunction differed according to the trial phase, but the rates in phase 1 trials were comparable, suggesting that those events tended to arise early in treatment. Fourth, the rates of these adverse events differed according to the ICI type and occurred more frequently in those who were treated with anti‐CTLA‐4 antibodies. Finally, we found that patients in the ICI groups were associated with a higher risk of serious‐grade and all‐grade pituitary‐adrenal dysfunction than those in the control groups. Most of these results were largely consistent with the findings of previous studies.^9^, ^10^, ^11^ Taken together, these findings indicate that ICI confers an elevated risk of pituitary‐adrenal dysfunction.

We chose to combine data that compared ICIs with placebo or other active regimens in one set of analyses because the comparator drugs seem to have no effect on these adverse events. In our analysis, a small proportion of the included studies examined the effects of ICI dosage regimens that were used as first‐line therapies or other combination therapies that were not approved for use. However, little heterogeneity was observed for the relative risks, despite the varied use of background treatments and differential use of comparator drugs. Notably, heterogeneity in the rates of these adverse events was observed across drugs, which might in part be attributable to the differential use of ICIs and might also be attributable to the varied phases of the clinical trials.

Previous studies have reported that the overall mean incidence of ICI‐associated adverse events did not differ between different cancer types^10^; therefore, we did not perform subgroup analyses based on particular cancer types to calculate whether specific pituitary‐adrenal dysfunction was more common in certain cancer types. However, we found that CTLA‐4 inhibitors appeared to have a higher rate of all‐grade and serious‐grade adverse events of the adverse events studied than PD‐1 and PD‐L1 inhibitors. The explanation for these differences might be attributed to the mechanistic underpinnings of each target. Unlike the PD‐1/PD‐L1 axis, which activates restricted subsets of T cells in the tumor microenvironment and in circulation, CTLA‐4 inhibition induces the breadth of T cell activation,^174^, ^175^ which is thought to be responsible for the high rate of immune‐based toxic effects during treatment or even long after treatment cessation.

Less is known about ICI‐induced hypopituitarism. Although our study found only 71 cases of serous‐grade hypopituitarism among 12 336 patients (0.58%), 50 cases occurred with anti‐CTLA‐4 therapy among 5790 patients. Likewise, among 45 cases of any‐grade hypopituitarism, 36 occurred with anti‐CTLA‐4 therapy among 1836 patients (1.96%). We did not make statistical inferences due to the small number of events. However, the risk of hypopituitarism was significantly higher in patients treated with ICI regimens than in those in the control group. Notably, few case reports for this event have been published in clinical trials, but hypopituitarism seemed higher in the spectrum of ICI‐induced endocrinopathies in cancer patients based on the scoping review of case reports.^8^

Our study has some limitations. First, because of the heterogeneity of the included studies, an assessment of additional potential risk factors, including the role of sex, age, and baseline pituitary‐adrenal function, is needed. However, some comparisons were limited by a lack of detailed clinical data for these uncommon safety outcomes. Considering the higher rate of these adverse events among patients on anti‐CTLA‐4 therapy, the second limitation is that the associations of clinical response, long‐term durability and irAEs with anti‐CTLA‐4 have not been established. Data from early trials have described that anti‐CTLA4‐related irAEs might be associated with clinical benefit.^176^, ^177^ A pooled analysis of 343 patients treated with ipilimumab showed that there was a trend toward superior disease control in patients with at least grade 2 irAEs when compared withthose with grade 1 irAEs.^177^ Patients with hypophysitis had a prolonged median survival time compared with those without hypophysitis following CTLA‐4 inhibitor therapy.^176^ Thus, anti‐CTLA‐4‐associated pituitary‐adrenal dysfunction might be suggestive of benefit rather than harm in certain conditions. The main limitation of our study is that the rates of these events were low, but there were still data derived from a small number of studies. A substantial amount of additional data is still needed to draw firm conclusions.

Although pituitary‐adrenal dysfunction associated with ICIs are rare, these toxicities can be severe or even life threatening with a higher relative risk than other oncologic interventions. As the use of ICIs increases in the clinic, these occurrences will become more common. Moreover, these occurrences tend to differ among various regimens and phases of trials. Additional information, such as baseline pituitary‐adrenal function, will be crucial to separate patients into subgroups that allow the identification of patients who are at the greatest risk of serious‐grade pituitary‐adrenal dysfunction.

## CONFLICT OF INTEREST

The authors declare that there is no duality of interest associated with this manuscript.
