# Supplementary material for: Immune checkpoint inhibitor‐associated pituitary‐adrenal dysfunction: A systematic review and meta‐analysis
Source: Cancer Med. 2019 Nov 3;8(18):7503–15. doi: 10.1002/cam4.2661 (PMC6912062; doi:10.1002/cam4.2661)
Supplement: Supplementary file 1 [file CAM4-8-7503-s001.pdf]

Supplementary

Table S1 Search strategy.

Data source	Search terms
**PubMed**	ALL FIELDS (ipilimumab OR MDX-010 OR tremelimumab OR CP-675,206 OR nivolumab OR BMS-963558 OR pembrolizumab OR MK-3475 OR atezolizumab OR MPDL3280A OR avelumab OR MSB0010718C OR durvalumab OR MEDI4736 OR cemiplimab OR REGN2810 OR toripalimab OR JS001 OR sintilimab OR IBI308) AND (phase)
**Embase**	ALL FIELDS ('phase '/exp OR 'phase') AND ('ipilimumab'/exp OR 'ipilimumab' OR 'MDX-010'/exp OR 'MDX-010' OR 'tremelimumab'/exp OR 'tremelimumab' OR 'CP-675,206 '/exp OR 'CP-675,206' OR 'nivolumab '/exp OR 'nivolumab' OR 'BMS-963558'/exp OR 'BMS-963558' OR 'pembrolizumab'/exp OR 'pembrolizumab' OR 'MK-3475'/exp OR 'MK-3475' OR 'atezolizumab'/exp OR 'atezolizumab' OR 'MPDL3280A'/exp OR 'MPDL3280A' OR 'avelumab'/exp OR 'avelumab' OR 'MSB0010718C'/exp OR 'MSB0010718C' OR 'pembrolizumab'/exp OR 'pembrolizumab' OR 'MK-3475'/exp OR 'MK-3475' OR 'atezolizumab'/exp OR 'atezolizumab' OR 'MPDL3280A'/exp OR 'MPDL3280A' OR 'avelumab'/exp OR 'avelumab' OR 'MSB0010718C'/exp OR 'MSB0010718C' OR 'durvalumab'/exp OR 'durvalumab' OR 'MEDI4736'/exp OR 'MEDI4736' OR 'cemiplimab'/exp OR 'cemiplimab' OR 'REGN2810'/exp OR 'REGN2810' OR 'toripalimab'/exp OR 'toripalimab' OR 'JS001'/exp OR 'JS001' OR 'sintilimab'/exp OR 'sintilimab' OR 'IBI308'/exp OR 'IBI308')
**ClinicalTrials.gov**	(ipilimumab OR MDX-010 OR tremelimumab OR CP-675,206 OR nivolumab OR BMS-963558 OR pembrolizumab OR MK-3475 OR atezolizumab OR MPDL3280A OR avelumab OR MSB0010718C OR durvalumab OR MEDI4736 OR cemiplimab OR REGN2810 OR toripalimab OR JS001 OR sintilimab OR IBI308)
**Cochrane library**	ALL TEXT ipilimumab OR MDX-010 OR tremelimumab OR CP-675,206 OR nivolumab OR BMS-963558 OR pembrolizumab OR MK-3475 OR atezolizumab OR MPDL3280A OR avelumab OR MSB0010718C OR durvalumab OR MEDI4736 OR cemiplimab OR REGN2810 OR toripalimab OR JS001 OR sintilimab OR IBI308

Table S2 Characteristics of eligible studies in patients with cancer receiving immune checkpoint inhibitors

No.	Year Author	International study	No. of countries involved	No. of study sites	Study phase	No. group	No. patients	No (%) Male	Cancer type	ICI type
1	2005 Ribas(1)	No	1	2	1		39	27 (69)	Mixed cancer types	Tremelimumab
2	2007 Yang(2)	NR	NR	NR	2	2	61	45(74)	Renal cellcancer	Ipilimumab
3	2008 Weber(3)	NR	NR	NR	1/2	3	88	57(65)	Melanoma	Ipilimumab
4	2009 Fong(4)	NR	NR	NR	1	1	24	24(100)	prostate cancer	Ipilimumab
5	2009 Ribas(5)	No	1	1	1	1	16	11(69)	Melanoma	Tremelimumab
6	2010 Chung(6)	NR	NR	NR	2	1	47	29(59)	Colorectal Cancer	Tremelimumab
7	2010 Hersh(7)	No	1	9	2	2	72	47(65)	Melanoma	Ipilimumab
8	2010 Hodi(8)	Yes	13	125	3	3	676	401(59)	Melanoma	Ipilimumab
9	2010 Kirkwood(9)	NR	NR	NR	2	1	251	151 (60)	Melanoma	Tremelimumab
10	2010 Royal(10)	NR	NR	NR	2	1	37	15(40)	Pancreatic cancer	Ipilimumab
11	2010 Sarnaik(11)	NR	NR	NR	2	1	75	44(59)	Melanoma	Ipilimumab
12	2010 Wolchok(12)	Yes	12	66	2	3	217	194(89)	Melanoma	Ipilimumab
13	2011 Robert(13)	Yes	23	127	3	2	502	301(60)	Melanoma	Ipilimumab
14	2012 Eertwegh(14)	No	1	1	1	1	28	NR	Prostate cancer	Ipilimumab
15	2012 Giacomo(15)	No	1	7	2	1	86	60(70)	Melanoma	Ipilimumab
16	2012 Lynch(16)	Yes	NR	NR	2	3	204	151(74)	NSCLC	Ipilimumab
17	2012 Madan(17)	No	1	1	1	1	30	NR	Prostate cancer	Ipilimumab
18	2012 Topalian(18)	Yes	1	13	1	5	306	203(66)	Mixed cancer types	Nivolumab
19	2013 Le(19)	No	1	1	1b	2	30	21(70)	Pancreatic cancer	Ipilimumab
20	2013 Millward(20)	NR	NR	NR	1	1	21	14(67)	Mixed cancer types	Tremelimumab
21	2013 Ribas(21)	Yes	21	144	3	2	655	372(57)	Melanoma	Tremelimumab
22	2013 Weber(22)	No	1	1	1	6	90	58(64)	Melanoma	Nivolumab
23	2013 Wolchok(23)	No	1	4	1	2	86	50(58)	Melanoma	Nivolumab Ipilimumab
24	2014 Kwon(24)	Yes	191	26	3	2	799	799(100)	Prostate cancer	Ipilimumab
25	2014 Robert(25)	Yes	4	NR	1	2	173	105(61)	Melanoma	Pembrolizumab
26	2015 Eggermont(26)	Yes	19	91	3	2	951	589(62)	Melanoma	Ipilimumab
27	2015 Herbst(27)	Yes	24	202	2/3	3	1033	634(61)	NSCLC	Pembrolizumab
28	2015 Horinouchi(28)	No	1	1	1	2	15	12(80)	NSCLC	Ipilimumab
29	2015 Le(29)	No	1	8	2	1	41	24(58)	Mixed cancer types	Pembrolizumab
30	2015 Postow(30)	Yes	2	21	3	2	142	95(67)	Melanoma	Nivolumab Ipilimumab
31	2015 Ribas(31)	Yes	12	73	2	3	540	327(60)	Melanoma	Pembrolizumab
32	2015 Rizvi(32)	Yes	4	27	2	1	117	85(72)	NSCLC	Nivolumab
33	2015 Robert(33)	Yes	16	NR	3	3	834	497(60)	Melanoma	Pembrolizumab Ipilimumab

34	2015 Weber(34)	Yes	14	90	3	2	405	261(64)	Melanoma	Nivolumab
35	2015 Yamazaki(35)	No	1	6	2	1	20	10 (50)	Melanoma	Ipilimumab
36	2015 Yamazaki(36)	No	1	6	2	1	15	10 (67)	Melanoma	Ipilimumab
37	2015 Zimmer(37)	NR	NR	NR	2	1	103	66(64)	Melanoma	Ipilimumab
38	2015 Zimmer(38)	NR	NR	NR	2	1	53	23(43)	Uveal Melanoma	Ipilimumab
39	2016 Antonia(39)	Yes	6	23	1/2	3	213	128(60)	SCLC	Nivolumab Ipilimumab
40	2016 Armand(40)	NR	NR	NR	1b	1	31	18 (58)	Hodgkin lymphoma	Pembrolizumab
41	2016 Brohl(41)	No	1	1	1b	1	31	18 (58)	Melanoma	Ipilimumab
42	2016 Hellmann(42)	No	1	8	1	2	77	41(53)	NSCLC	Nivolumab Ipilimumab
43	2016 Reck(43)	Yes	34	224	3	2	954	643(67)	SCLC	Ipilimumab
44	2016 Reck(44)	Yes	16	142	3	3	305	187(61)	NSCLC	Pembrolizumab
45	2016 Ribas(45)	Yes	4		1b	1	655	405 (62)	Melanoma	Pembrolizumab
46	2016 Seiwert(46)	NR	NR	NR	1b	1	60	49 (82)	HNSC	Pembrolizumab
47	2016 Subudhi(47)	NO	1	1	2	1	27	NR	Prostate cancer	Ipilimumab
48	2016 Weber(48)	No	1	1	0.5	1	92	60(65)	Melanoma	Nivolumab
49	2016 Weber(49)	No	1	9	2	2	138	92(67)	Melanoma	Nivolumab Ipilimumab
50	2016 Wilgenhof(50)	No	1	1	2	1	39	23 (59)	Melanoma	Ipilimumab
51	2017 Ascierto(51)	Yes	21	87	3	2	727	450(62)	Melanoma	Ipilimumab
52	2017 Badros(52)	No	1	1	2	1	48	31 (65)	Myeloma	Pembrolizumab
53	2017 Bellmunt(53)	Yes	29	120	3	2	542	NR	Urothelial carcinoma	Pembrolizumab
54	2017 Callahan(54)	No	1	4	1	1	94	50 (53)	Melanoma	Nivolumab Ipilimumab
55	2017 El-Khoueiry(55)	Yes	4 11	7 39	1/2	2	262	NR	HCC	Nivolumab
56	2017 Galsky(56)	Yes	1	8	2	1	36	29 (81)	Urothelial cancer	Ipilimumab
57	2017 Govindan(57)	Yes	36	285	3	2	749	635(85)	NSCLC	Ipilimumab
58	2017 Gulley(58)	No	1	58	1	1	184	100 (54)	NSCLC	Avelumab
59	2017 Hui(59)	NR	NR	NR	1	1	101	60 (59)	NSCLC	Pembrolizumab
60	2017 Kang(60)	Yes	3	49	3	2	493	348(70)	GEJ	Nivolumab
61	2017 Long(61)	Yes	3	12	1b	1	153	101 (66)	Melanoma	Pembrolizumab Ipilimumab
62	2017 Overman(62)	Yes	8	31	2	1	74	44 (59)	Colorectal cancer	Nivolumab
63	2017 Patel(63)	Yes	10	80	1	1	249	178 (72)	Urothelial carcinoma	Avelumab
64	2017 Tang(64)	No	1	1	1	1	35	18(51)	Mixed cancer types	Ipilimumab
65	2017 Tawbi(65)	No	1	12	2	2	84	53(63)	Sarcoma	Pembrolizumab
66	2017 Tolcher(66)	No	1	9	1b	1	23	14(61)	Mixed cancer types	Pembrolizumab
67	2017 Weber(67)	Yes	25	130	3	2	906	527(58)	Melanoma	Nivolumab Ipilimumab
68	2017 Williams(68)	No	1	2	1	2	16	12(75)	Melanoma	Ipilimumab
69	2017 Yamazaki(69)	NR	NR	NR	1b	1	42	26 (62)	Melanoma	Pembrolizumab
70	2017 Yi(70)	No	1	1	2	1	24	12 (50)	NSCLC	Ipilimumab
71	2018 Adams(71)	Yes	9	27	2	1	84	0	Breast Cancer	Pembrolizumab
72	2018 Amaria(72)	No	1	1	2	2	23	NR	Melanoma	Iipilimumab Nivolumab

73	2018 Ariyan(73)	No	1	1	2	1	26	16 (62)	Melanoma	Ipilimumab
74	2018 Arkenau(74)	Yes	NR	NR	1	1	26	8 (31)	Biliary tract cancer	Pembrolizumab
75	2018 Armand(75)	Yes	10	34	2	1	243	141 (58)	Hodgkin lymphoma	Nivolumab
76	2018 Atkins(76)	NR	NR	NR	1b	2	39	25(64)	Mixed cancer types	Pembrolizumab Ipilimumab
77	2018 Atkins(77)	No	1	10	1b	1	52	41 (79)	Renal cell cancer	Pembrolizumab
78	2018 Bajor(78)	No	1	1	1	1	24	11 (46)	Melanoma	Tremelimumab
79	2018 Balar(79)	Yes	20	91	2	1	370	286 (77)	urothelial cancer	Pembrolizumab
80	2018 Barta(80)	No	1	5	2	1	18	8 (47)	Mature T-cell lymphoma	Pembrolizumab
81	2018 Boudadi(81)	No	1	1	2	1	15	NR	Prostate cancer	Ipilimumab Nivolumab
82	2018 Choueiri(82)	Yes	3	14	1b	1	55	42 (76)	CCRCC	Avelumab
83	2018 Chung(83)	No	1	1	1	1	11	4(36)	Mixed cancer types	Pembrolizumab
84	2018 D'Angelo(84)	No	1	15	2	2	85	41(48)	Sarcoma	Nivolumab Ipilimumab
85	2018 Davar(85)	No	1	1	1b /2	1	43	26 (60)	Melanoma	Pembrolizumab
86	2018 Doi(86)	No	1	1	1b	1	23	19 (83)	GEJ	Pembrolizumab
87	2018 Eggermont(87)	Yes	23	123	3	2	1019	628(62)	Melanoma	Pembrolizumab
88	2018 Fehrenbacher(88)	Yes	31	218	3	2	1225	NR	NSCLC	Atezolizumab
89	2018 Gadgeel(89)	NR	NR	NR	1	3	74	37(50)	NSCLC	Pembrolizumab
90	2018 Gandhi(90)	Yes	NR	NR	3	2	616	363(59)	NSCLC	Pembrolizumab
91	2018 Garassino(91)	Yes	18	139	2	3	444	242(54)	NSCLC	Durvalumab
92	2018 Gubens(92)	NR	NR	NR	1/2	2	51	26(51)	NSCLC	Pembrolizumab Ipilimumab
93	2018 Haag(93)	No	1	1	2	1	25	20 (80)	melanoma	Ipilimumab
94	2018 Hodi(94)	Yes	21	137	3	3	945	610(64)	Melanoma	Nivolumab Ipilimumab
95	2018 Janjigian(95)	Yes	6	18	1/2	3	160	124(78)	GEJ	Nivolumab Ipilimumab
96	2018 Lisberg(96)	No	1	1	2	1	25	NR	NSCLC	Pembrolizumab
97	2018 Liu(97)	No	1	11	1b	3	76	40(53)	NSCLC	Atezolizumab
98	2018 Long(98)	No	1	4	2	3	76	59(78)	Melanoma	Nivolumab Ipilimumab
99	2018 Namikawa(99)	No	1	NR	2	1	30	NR	Melanoma	Nivolumab Ipilimumab
100	2018 Ott(100)	NR	NR	NR	1b	1	475	194(41)	Mixed cancer types	Pembrolizumab
101	2018 Paz-Ares(101)	Yes	17	137	3	2	559	455(81)	NSCLC	Pembrolizumab
102	2018 Ribas(102)	NR	NR	NR	1b	1	22	15 (68)	Melanoma	Pembrolizumab
103	2018 Sakamuri(103)	No	1	1	1		36	12 (33)	Mixed cancer types	Ipilimumab
104	2018 Shitara(104)	Yes	30	148	3	2	592	410(69)	GEJ	Pembrolizumab
105	2018 Tarhini(105)	No	1	NR	2	4	81	48(59)	Melanoma	Ipilimumab
106	2018 Tawbi(106)	No	1	28	2	1	94	65 (69)	Melanoma	Nivolumab Ipilimumab
107	2018 Tourneau(107)	Yes	NR	NR	1b	1	50	24 (48)	Adrenocortical carcinoma	Avelumab
108	2018 Yang(108)	No	1	1	2	1	24	12 (50)	NSCLC	Ipilimumab
109	2018 Zhu(109)	Yes	10	47	2	1	104	86 (83)	HCC	Pembrolizumab

110	2019 Chung(110)	Yes	NR	NR	1b	2	150	114(76)	GEJ	Avelumab
111	2019 Chung(111)	Yes	NR	NR	2	1	98	NR	Cervical cancer	Pembrolizumab
112	2019 Disselhorst(112)	No	1	1	2	1	35	27 (77)	pleural mesothelioma	Ipilimumab Nivolumab
113	2019 Emens (113)	Yes	4	21	1	1	113	NR	Breast Cancer	Atezolizumab
114	2019 Fountain(114)	No	1	1	1/2	1	10	NR	Uveal melanoma	Ipilimumab
115	2019 Katsuya(115)	No	NR	NR	2	1	15	12(80)	Thymic carcinoma	Nivolumab
116	2019 Leighl(116)	NR	NR	NR	1	2	550	289(52)	NSCLC	Pembrolizumab
117	2019 Loibl(117)	No	1	1	2	2	174	NR	Breast cancer	Durvalumab
118	2019 Makker(118)	No	1	11	1	1	53	NR	Endometrial cancer	Pembrolizumab
119	2019 Matulonis(119)	NR	NR	NR	2	1	376	NR	Ovarian cancer	Pembrolizumab
120	2019 Mok(120)	Yes	32	213	3	2	1274	902(71)	NSCLC	Pembrolizumab
121	2019 Nghiem(121)	No	1	13	2	1	50	34 (68)	Merkel cell carcinoma	Pembrolizumab
122	2019 Scherpereel(122)	No	1	21	2	2	124	100(81)	Pleural mesothelioma	Nivolumab Ipilimumab
123	NCT00162123(123)	Yes	10	58	2	8	242	157(65)	Melanoma	Ipilimumab
124	NCT00323882(124)	No	1	14	1/2	5	71	71(100)	Prostate Cancer	Ipilimumab
125	NCT00527735(125)	Yes	8	72	2	3	334	249(74)	NSCLC/SCLC	Ipilimumab
126	NCT00623766(126)	No	1	14	2	2	72	44(61)	Melanoma	Ipilimumab
127	NCT00796991(127)	No	1	4	2	3	59	38(64)	Melanoma	Ipilimumab
128	NCT00920907(128)	No	1	7	1	2	75	49(65)	Melanoma	Ipilimumab
129	NCT01057810(129)	Yes	24	134	3	2	602	602(100)	Prostate Cancer	Ipilimumab
130	NCT01134614(130)	No	1	258	2	2	245	163(66)	Melanoma	Ipilimumab
131	NCT01354431(131)	Yes	4	41	2	3	168	121(72)	CCRCC	Nivolumab
132	NCT01498978(132)	No	1	2	1	1	10	10(100)	Prostate Cancer	Ipilimumab
133	NCT01585987(133)	Yes	12	36	2	2	114	77(68)	GEJ	Ipilimumab
134	NCT01611558(134)	No	1	17	2	1	40	0(0)	Ovarian cancer	Ipilimumab
135	NCT01668784(135)	Yes	24	156	3	2	821	619(75)	CCRCC	Nivolumab
136	NCT01673854(136)	No	1	14	2	1	46	NR	Melanoma	Ipilimumab
137	NCT01673867(137)	Yes	22	134	3	2	582	319(55)	NSCLC	Nivolumab
138	NCT01708941(138)	No	1	282	2	4	81	48(59)	Melanoma	Ipilimumab
139	NCT01721772(139)	Yes	16	75	3	2	418	246(59)	Melanoma	Nivolumab
140	NCT01740297(140)	Yes	3	33	1/2	3	217	125(58)	Melanoma	Ipilimumab
141	NCT01843374(141)	Yes	19	104	3	2	571	434(76)	Mesothelioma	Tremelimumab
142	NCT01984242(142)	Yes	7	57	2	3	305	230(75)	Renal Cell Carcinoma	Atezolizumab
143	NCT02031458(143)	Yes	19	111	2	3	659	388(59)	NSCLC	Atezolizumab
144	NCT02041533(144)	Yes	26	141	3	2	541	271(50)	NSCLC	Nivolumab
145	NCT02105636(145)	Yes	15	64	3	2	361	300(83)	HNSC	Nivolumab
146	NCT02231749(146)	Yes	28	175	3	2	1096	808(74)	Renal cell carcinoma	Nivolumab Ipilimumab
147	NCT02250326(147)	Yes	7	34	2	3	240	154(64)	NSCLC	Durvalumab

148	NCT02255097(148)	NR	NR	NR	2	1	171	138(81)	HNSC	Pembrolizumab
149	NCT02319044(149)	Yes	15	127	2	3	267	220(82)	HNSC	Tremelimumab
150	NCT02352948(150)	Yes	26	203	3	6	595	398(67)	NSCLC	Durvalumab Tremelimumab
151	NCT02367781(151)	Yes	9	156	3	2	723	415(57)	NSCLC	Atezolizumab
152	NCT02387996(152)	Yes	11	67	2	1	270	211(78)	Bladder Cancer	Nivolumab
153	NCT02395172(153)	Yes	29	260	3	2	792	542(68)	NSCLC	Avelumab
154	NCT02420821(154)	Yes	21	165	3	2	915	669(73)	Renal cell carcinoma	Atezolizumab
155	NCT02527434(155)	Yes	5	14	2	3	64	37(58)	Mixed cancer types	Durvalumab Tremelimumab
156	NCT02685826(156)	Yes	8	32	1/2	3	56	37(66)	Myeloma	Durvalumab
157	NCT02714218(157)	Yes	13	57	3	3	385	222(58)	Melanoma	Nivolumab Ipilimumab
158	NCT02752074(158)	Yes	23	135	3	2	706	423(60)	Melanoma	Pembrolizumab
159	NCT02905266(159)	Yes	4	16	3	2	106	62(58)	Melanoma	Nivolumab Ipilimumab
160	NCT03029780(160)	Yes	3	11	2	2	104	50(48)	Renal cell carcinoma	Nivolumab Ipilimumab

Abbreviations: BLCA, Bladder urothelial Carcinoma; CCRCC, Clear-cell renal cell carcinoma; GEJ, Gastric or gastro-oesophageal junction cancer; HCC, Hepatocellular carcinoma; HNSC, Head and neck squamous cell carcinoma; NSCLC, Non-small-cell lung cancer; SCLC, Small-cell lung cancer.

Table S3 Publication bias test for rates of pituitary-adrenal dysfunction

Outcomes	Egger's test
All-grade adrenal insufficiency	<0.001
Serious-grade adrenal insufficieny	<0.001
All-grade hypophysitis	<0.01
Serious-grade hypophysitis	<0.001

Table S4 Publication bias test for risk of pituitary-adrenal dysfunction based on clinical controlled trials

Outcomes	Egger's test	Begg's test
All-grade adrenal insufficiency	0.094	0.533
Serious-grade adrenal insufficieny	0.253	0.802
All-grade hypophysitis	0.001	0.049
Serious-grade hypophysitis	0.329	0.027
All-grade hypopituitarism	0.501	1.000
Serious-grade hypopituitarism	0.000	0.013

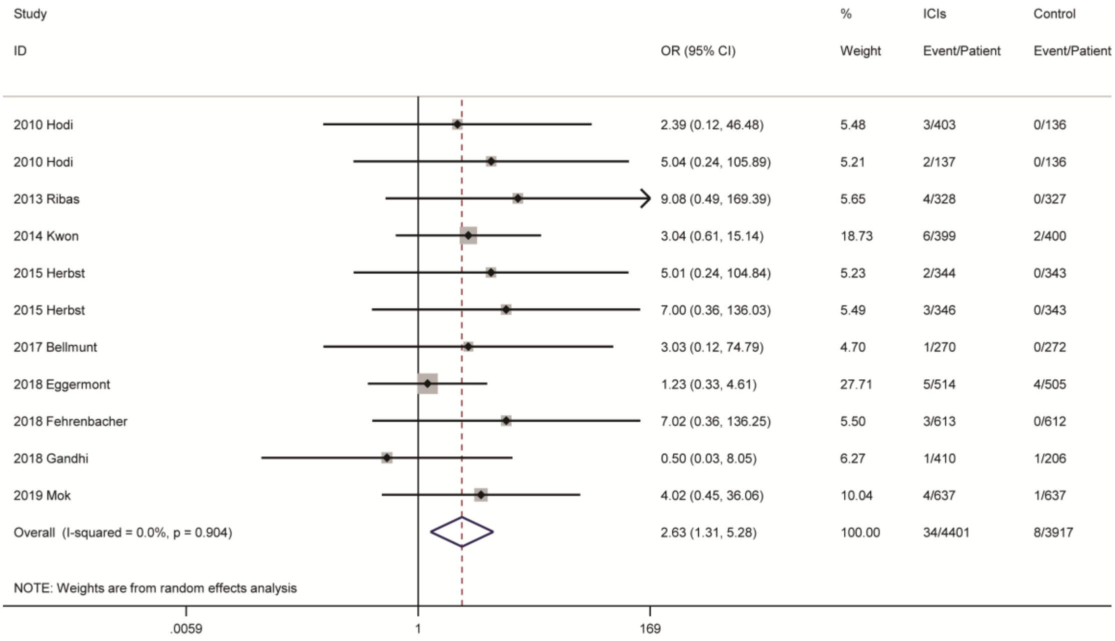

Figure S1 Risk of all-grade adrenal insufficiency in patients treated with ICIs versus control

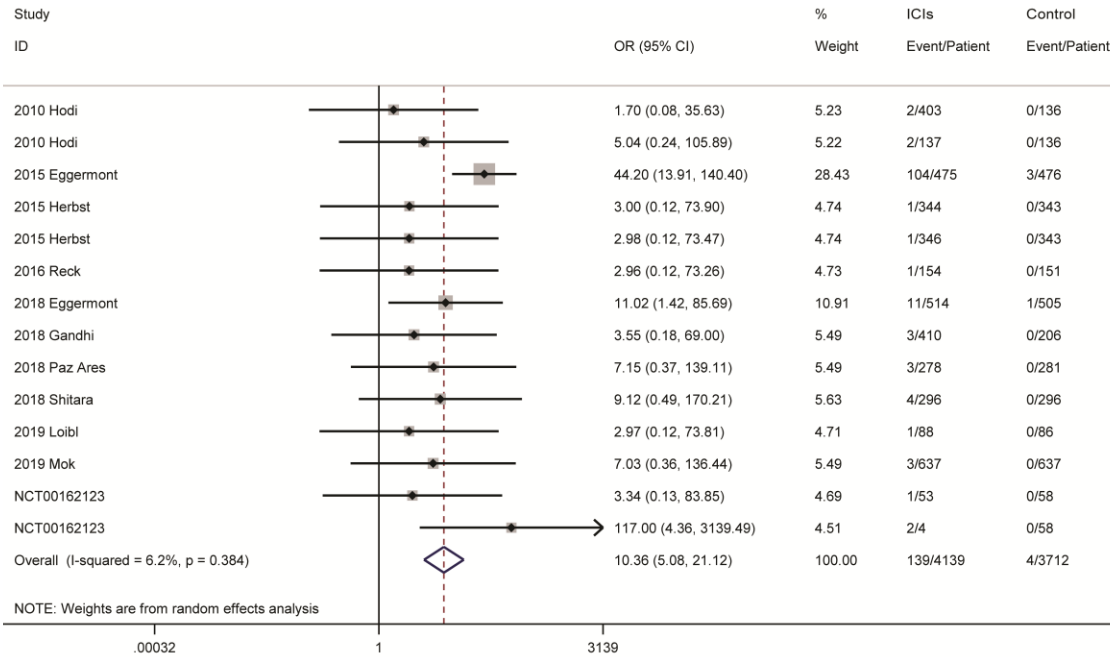

Figure S2 Risk of all-grade hypophysitis in patients treated with ICIs versus control

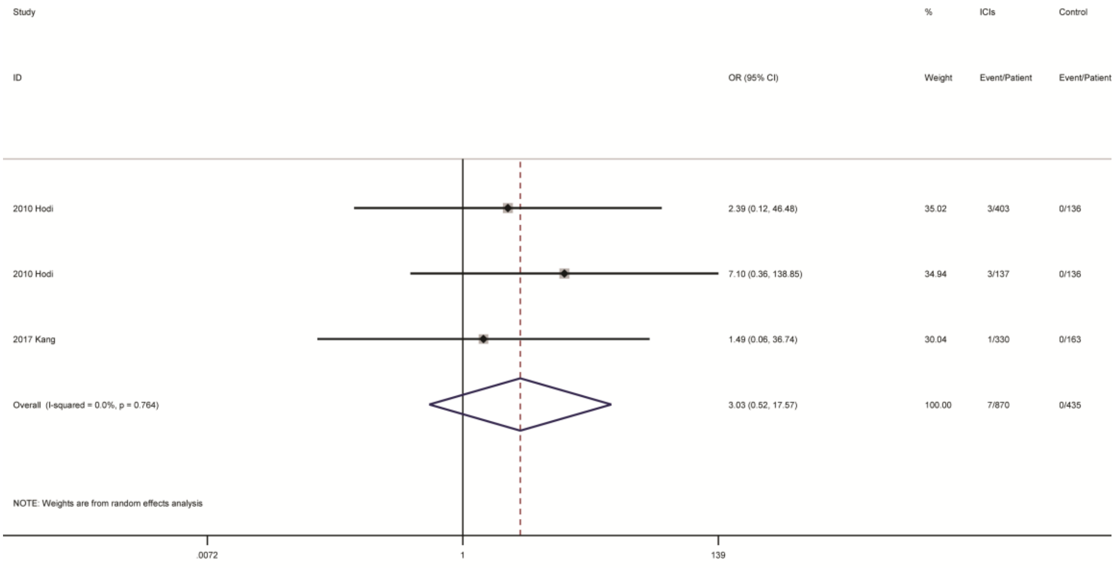

Figure S3 Risk of all-grade hypopituitarism in patients treated with ICIs versus control

References

1. Ribas A, Camacho LH, Lopez-Berestein G, Pavlov D, Bulanhagui CA, Millham R, et al. Antitumor activity in melanoma and anti-self responses in a phase I trial with the anti-cytotoxic T lymphocyte-associated antigen 4 monoclonal antibody CP-675,206. J Clin Oncol. 2005;23(35):8968-77. Epub 2005/10/06.
2. Yang JC, Hughes M, Kammula U, Royal R, Sherry RM, Topalian SL, et al. Ipilimumab (anti-CTLA4 antibody) causes regression of metastatic renal cell cancer associated with enteritis and hypophysitis. J Immunother. 2007;30(8):825-30. Epub 2007/12/01.
3. Weber JS, O'Day S, Urba W, Powderly J, Nichol G, Yellin M, et al. Phase I/II study of ipilimumab for patients with metastatic melanoma. J Clin Oncol. 2008;26(36):5950-6. Epub 2008/11/20.
4. Fong L, Kwek SS, O'Brien S, Kavanagh B, McNeel DG, Weinberg V, et al. Potentiating endogenous antitumor immunity to prostate cancer through combination immunotherapy with CTLA4 blockade and GM-CSF. Cancer Res. 2009;69(2):609-15. Epub 2009/01/17.
5. Ribas A, Comin-Anduix B, Chmielowski B, Jalil J, de la Rocha P, McCannel TA, et al. Dendritic cell vaccination combined with CTLA4 blockade in patients with metastatic melanoma. Clin Cancer Res. 2009;15(19):6267-76. Epub 2009/10/01.
6. Chung KY, Gore I, Fong L, Venook A, Beck SB, Dorazio P, et al. Phase II study of the anti-cytotoxic T-lymphocyte-associated antigen 4 monoclonal antibody, tremelimumab, in patients with refractory metastatic colorectal cancer. J Clin Oncol. 2010;28(21):3485-90. Epub 2010/05/26.
7. Hersh EM, O'Day SJ, Powderly J, Khan KD, Pavlick AC, Cranmer LD, et al. A phase II multicenter study of ipilimumab with or without dacarbazine in chemotherapy-naive patients with

advanced melanoma. Invest New Drugs. 2011;29(3):489-98. Epub 2010/01/19.
8. Hodi FS, O'Day SJ, McDermott DF, Weber RW, Sosman JA, Haanen JB, et al. Improved survival with ipilimumab in patients with metastatic melanoma. N Engl J Med. 2010;363(8):711-23. Epub 2010/06/08.
9. Kirkwood JM, Lorigan P, Hersey P, Hauschild A, Robert C, McDermott D, et al. Phase II trial of tremelimumab (CP-675,206) in patients with advanced refractory or relapsed melanoma. Clin Cancer Res. 2010;16(3):1042-8. Epub 2010/01/21.
10. Royal RE, Levy C, Turner K, Mathur A, Hughes M, Kammula US, et al. Phase 2 trial of single agent Ipilimumab (anti-CTLA-4) for locally advanced or metastatic pancreatic adenocarcinoma. J Immunother. 2010;33(8):828-33. Epub 2010/09/16.
11. Sarnaik AA, Yu B, Yu D, Morelli D, Hall M, Bogle D, et al. Extended dose ipilimumab with a peptide vaccine: immune correlates associated with clinical benefit in patients with resected high-risk stage IIIc/IV melanoma. Clin Cancer Res. 2011;17(4):896-906. Epub 2010/11/26.
12. O'Day SJ, Maio M, Chiarion-Sileni V, Gajewski TF, Pehamberger H, Bondarenko IN, et al. Efficacy and safety of ipilimumab monotherapy in patients with pretreated advanced melanoma: a multicenter single-arm phase II study. Annals of oncology : official journal of the European Society for Medical Oncology / ESMO. 2010;21(8):1712-7. Epub 2010/02/12.
13. Robert C, Thomas L, Bondarenko I, O'Day S, Weber J, Garbe C, et al. Ipilimumab plus dacarbazine for previously untreated metastatic melanoma. N Engl J Med. 2011;364(26):2517-26. Epub 2011/06/07.
14. van den Eertwegh AJ, Versluis J, van den Berg HP, Santegoets SJ, van Moorselaar RJ, van der Sluis TM, et al. Combined immunotherapy with granulocyte-macrophage colony-stimulating factor-transduced allogeneic prostate cancer cells and ipilimumab in patients with metastatic castration-resistant prostate cancer: a phase 1 dose-escalation trial. Lancet Oncol. 2012;13(5):509-17. Epub 2012/02/14.
15. Di Giacomo AM, Ascierto PA, Pilla L, Santinami M, Ferrucci PF, Giannarelli D, et al. Ipilimumab and fotemustine in patients with advanced melanoma (NIBIT-M1): an open-label, single-arm phase 2 trial. Lancet Oncol. 2012;13(9):879-86. Epub 2012/08/17.
16. Lynch TJ, Bondarenko I, Luft A, Serwatowski P, Barlesi F, Chacko R, et al. Ipilimumab in combination with paclitaxel and carboplatin as first-line treatment in stage IIIB/IV non-small-cell lung cancer: results from a randomized, double-blind, multicenter phase II study. J Clin Oncol. 2012;30(17):2046-54. Epub 2012/05/02.
17. Madan RA, Mohebtash M, Arlen PM, Vergati M, Rauckhorst M, Steinberg SM, et al. Ipilimumab and a poxviral vaccine targeting prostate-specific antigen in metastatic castration-resistant prostate cancer: a phase 1 dose-escalation trial. Lancet Oncol. 2012;13(5):501-8. Epub 2012/02/14.
18. Topalian SL, Hodi FS, Brahmer JR, Gettinger SN, Smith DC, McDermott DF, et al. Safety, activity, and immune correlates of anti-PD-1 antibody in cancer. N Engl J Med. 2012;366(26):2443-54. Epub 2012/06/05.
19. Le DT, Lutz E, Uram JN, Sugar EA, Onners B, Solt S, et al. Evaluation of ipilimumab in combination with allogeneic pancreatic tumor cells transfected with a GM-CSF gene in previously treated pancreatic cancer. J Immunother. 2013;36(7):382-9. Epub 2013/08/09.
20. Millward M, Underhill C, Lobb S, McBurnie J, Meech SJ, Gomez-Navarro J, et al. Phase I study of tremelimumab (CP-675 206) plus PF-3512676 (CPG 7909) in patients with melanoma or advanced solid tumours. Br J Cancer. 2013;108(10):1998-2004. Epub 2013/05/09.

21. Ribas A, Kefford R, Marshall MA, Punt CJ, Haanen JB, Marmol M, et al. Phase III randomized clinical trial comparing tremelimumab with standard-of-care chemotherapy in patients with advanced melanoma. J Clin Oncol. 2013;31(5):616-22. Epub 2013/01/09.
22. Weber JS, Kudchadkar RR, Yu B, Gallenstein D, Horak CE, Inzunza HD, et al. Safety, efficacy, and biomarkers of nivolumab with vaccine in ipilimumab-refractory or -naive melanoma. J Clin Oncol. 2013;31(34):4311-8. Epub 2013/10/23.
23. Wolchok JD, Kluger H, Callahan MK, Postow MA, Rizvi NA, Lesokhin AM, et al. Nivolumab plus ipilimumab in advanced melanoma. N Engl J Med. 2013;369(2):122-33. Epub 2013/06/04.
24. Kwon ED, Drake CG, Scher HI, Fizazi K, Bossi A, van den Eertwegh AJ, et al. Ipilimumab versus placebo after radiotherapy in patients with metastatic castration-resistant prostate cancer that had progressed after docetaxel chemotherapy (CA184-043): a multicentre, randomised, double-blind, phase 3 trial. Lancet Oncol. 2014;15(7):700-12. Epub 2014/05/17.
25. Robert C, Ribas A, Wolchok JD, Hodi FS, Hamid O, Kefford R, et al. Anti-programmed-death-receptor-1 treatment with pembrolizumab in ipilimumab-refractory advanced melanoma: a randomised dose-comparison cohort of a phase 1 trial. Lancet. 2014;384(9948):1109-17. Epub 2014/07/19.
26. Eggermont AM, Chiarion-Sileni V, Grob JJ, Dummer R, Wolchok JD, Schmidt H, et al. Adjuvant ipilimumab versus placebo after complete resection of high-risk stage III melanoma (EORTC 18071): a randomised, double-blind, phase 3 trial. Lancet Oncol. 2015;16(5):522-30. Epub 2015/04/05.
27. Herbst RS, Baas P, Kim DW, Felip E, Perez-Gracia JL, Han JY, et al. Pembrolizumab versus docetaxel for previously treated, PD-L1-positive, advanced non-small-cell lung cancer (KEYNOTE-010): a randomised controlled trial. Lancet. 2016;387(10027):1540-50. Epub 2015/12/30.
28. Horinouchi H, Yamamoto N, Fujiwara Y, Sekine I, Nokihara H, Kubota K, et al. Phase I study of ipilimumab in phased combination with paclitaxel and carboplatin in Japanese patients with non-small-cell lung cancer. Invest New Drugs. 2015;33(4):881-9. Epub 2015/05/01.
29. Le DT, Uram JN, Wang H, Bartlett BR, Kemberling H, Eyring AD, et al. PD-1 Blockade in Tumors with Mismatch-Repair Deficiency. N Engl J Med. 2015;372(26):2509-20. Epub 2015/06/02.
30. Postow MA, Chesney J, Pavlick AC, Robert C, Grossmann K, McDermott D, et al. Nivolumab and ipilimumab versus ipilimumab in untreated melanoma. N Engl J Med. 2015;372(21):2006-17. Epub 2015/04/22.
31. Ribas A, Puzanov I, Dummer R, Schadendorf D, Hamid O, Robert C, et al. Pembrolizumab versus investigator-choice chemotherapy for ipilimumab-refractory melanoma (KEYNOTE-002): a randomised, controlled, phase 2 trial. Lancet Oncol. 2015;16(8):908-18. Epub 2015/06/28.
32. Rizvi NA, Mazieres J, Planchard D, Stinchcombe TE, Dy GK, Antonia SJ, et al. Activity and safety of nivolumab, an anti-PD-1 immune checkpoint inhibitor, for patients with advanced, refractory squamous non-small-cell lung cancer (CheckMate 063): a phase 2, single-arm trial. Lancet Oncol. 2015;16(3):257-65. Epub 2015/02/24.
33. Robert C, Schachter J, Long GV, Arance A, Grob JJ, Mortier L, et al. Pembrolizumab versus Ipilimumab in Advanced Melanoma. N Engl J Med. 2015;372(26):2521-32. Epub 2015/04/22.
34. Weber JS, D'Angelo SP, Minor D, Hodi FS, Gutzmer R, Neyns B, et al. Nivolumab versus chemotherapy in patients with advanced melanoma who progressed after anti-CTLA-4 treatment (CheckMate 037): a randomised, controlled, open-label, phase 3 trial. Lancet Oncol. 2015;16(4):375-84. Epub 2015/03/22.
35. Yamazaki N, Uhara H, Fukushima S, Uchi H, Shibagaki N, Kiyohara Y, et al. Phase II study of the

immune-checkpoint inhibitor ipilimumab plus dacarbazine in Japanese patients with previously untreated, unresectable or metastatic melanoma. Cancer chemotherapy and pharmacology. 2015;76(5):969-75. Epub 2015/09/27.
36. Yamazaki N, Kiyohara Y, Uhara H, Fukushima S, Uchi H, Shibagaki N, et al. Phase II study of ipilimumab monotherapy in Japanese patients with advanced melanoma. Cancer chemotherapy and pharmacology. 2015;76(5):997-1004. Epub 2015/09/28.
37. Zimmer L, Eigentler TK, Kiecker F, Simon J, Utikal J, Mohr P, et al. Open-label, multicenter, single-arm phase II DeCOG-study of ipilimumab in pretreated patients with different subtypes of metastatic melanoma. J Transl Med. 2015;13:351. Epub 2015/11/07.
38. Zimmer L, Vaubel J, Mohr P, Hauschild A, Utikal J, Simon J, et al. Phase II DeCOG-study of ipilimumab in pretreated and treatment-naive patients with metastatic uveal melanoma. PLoS One. 2015;10(3):e0118564. Epub 2015/03/12.
39. Antonia SJ, Lopez-Martin JA, Bendell J, Ott PA, Taylor M, Eder JP, et al. Nivolumab alone and nivolumab plus ipilimumab in recurrent small-cell lung cancer (CheckMate 032): a multicentre, open-label, phase 1/2 trial. Lancet Oncol. 2016;17(7):883-95. Epub 2016/06/09.
40. Armand P, Shipp MA, Ribrag V, Michot JM, Zinzani PL, Kuruvilla J, et al. Programmed Death-1 Blockade With Pembrolizumab in Patients With Classical Hodgkin Lymphoma After Brentuximab Vedotin Failure. J Clin Oncol. 2016;34(31):3733-9. Epub 2016/06/30.
41. Brohl AS, Khushalani NI, Eroglu Z, Markowitz J, Thapa R, Chen YA, et al. A phase IB study of ipilimumab with peginterferon alfa-2b in patients with unresectable melanoma. J Immunother Cancer. 2016;4:85. Epub 2016/12/30.
42. Hellmann MD, Rizvi NA, Goldman JW, Gettinger SN, Borghaei H, Brahmer JR, et al. Nivolumab plus ipilimumab as first-line treatment for advanced non-small-cell lung cancer (CheckMate 012): results of an open-label, phase 1, multicohort study. Lancet Oncol. 2017;18(1):31-41. Epub 2016/12/10.
43. Reck M, Luft A, Szczesna A, Havel L, Kim SW, Akerley W, et al. Phase III Randomized Trial of Ipilimumab Plus Etoposide and Platinum Versus Placebo Plus Etoposide and Platinum in Extensive-Stage Small-Cell Lung Cancer. J Clin Oncol. 2016;34(31):3740-8. Epub 2016/07/28.
44. Reck M, Rodriguez-Abreu D, Robinson AG, Hui R, Csoszi T, Fulop A, et al. Pembrolizumab versus Chemotherapy for PD-L1-Positive Non-Small-Cell Lung Cancer. N Engl J Med. 2016;375(19):1823-33. Epub 2016/10/11.
45. Ribas A, Hamid O, Daud A, Hodi FS, Wolchok JD, Kefford R, et al. Association of Pembrolizumab With Tumor Response and Survival Among Patients With Advanced Melanoma. Jama. 2016;315(15):1600-9. Epub 2016/04/20.
46. Seiwert TY, Burtness B, Mehra R, Weiss J, Berger R, Eder JP, et al. Safety and clinical activity of pembrolizumab for treatment of recurrent or metastatic squamous cell carcinoma of the head and neck (KEYNOTE-012): an open-label, multicentre, phase 1b trial. Lancet Oncol. 2016;17(7):956-65. Epub 2016/06/02.
47. Subudhi SK, Aparicio A, Gao J, Zurita AJ, Araujo JC, Logothetis CJ, et al. Clonal expansion of CD8 T cells in the systemic circulation precedes development of ipilimumab-induced toxicities. Proc Natl Acad Sci U S A. 2016;113(42):11919-24. Epub 2016/10/30.
48. Weber J, Gibney G, Kudchadkar R, Yu B, Cheng P, Martinez AJ, et al. Phase I/II Study of Metastatic Melanoma Patients Treated with Nivolumab Who Had Progressed after Ipilimumab. Cancer Immunol Res. 2016;4(4):345-53. Epub 2016/02/14.

49. Weber JS, Gibney G, Sullivan RJ, Sosman JA, Slingluff CL, Jr., Lawrence DP, et al. Sequential administration of nivolumab and ipilimumab with a planned switch in patients with advanced melanoma (CheckMate 064): an open-label, randomised, phase 2 trial. Lancet Oncol. 2016;17(7):943-55. Epub 2016/06/09.
50. Wilgenhof S, Corthals J, Heirman C, van Baren N, Lucas S, Kvistborg P, et al. Phase II Study of Autologous Monocyte-Derived mRNA Electroporated Dendritic Cells (TriMixDC-MEL) Plus Ipilimumab in Patients With Pretreated Advanced Melanoma. J Clin Oncol. 2016;34(12):1330-8. Epub 2016/03/02.
51. Ascierto PA, Del Vecchio M, Robert C, Mackiewicz A, Chiarion-Sileni V, Arance A, et al. Ipilimumab 10 mg/kg versus ipilimumab 3 mg/kg in patients with unresectable or metastatic melanoma: a randomised, double-blind, multicentre, phase 3 trial. Lancet Oncol. 2017;18(5):611-22. Epub 2017/04/01.
52. Badros A, Hyjek E, Ma N, Lesokhin A, Dogan A, Rapoport AP, et al. Pembrolizumab, pomalidomide, and low-dose dexamethasone for relapsed/refractory multiple myeloma. Blood. 2017;130(10):1189-97. Epub 2017/05/04.
53. Bellmunt J, de Wit R, Vaughn DJ, Fradet Y, Lee JL, Fong L, et al. Pembrolizumab as Second-Line Therapy for Advanced Urothelial Carcinoma. N Engl J Med. 2017;376(11):1015-26. Epub 2017/02/18.
54. Callahan MK, Kluger H, Postow MA, Segal NH, Lesokhin A, Atkins MB, et al. Nivolumab Plus Ipilimumab in Patients With Advanced Melanoma: Updated Survival, Response, and Safety Data in a Phase I Dose-Escalation Study. J Clin Oncol. 2018;36(4):391-8. Epub 2017/10/19.
55. El-Khoueiry AB, Sangro B, Yau T, Crocenzi TS, Kudo M, Hsu C, et al. Nivolumab in patients with advanced hepatocellular carcinoma (CheckMate 040): an open-label, non-comparative, phase 1/2 dose escalation and expansion trial. Lancet. 2017;389(10088):2492-502. Epub 2017/04/25.
56. Galsky MD, Wang H, Hahn NM, Twardowski P, Pal SK, Albany C, et al. Phase 2 Trial of Gemcitabine, Cisplatin, plus Ipilimumab in Patients with Metastatic Urothelial Cancer and Impact of DNA Damage Response Gene Mutations on Outcomes. European urology. 2018;73(5):751-9. Epub 2017/12/19.
57. Govindan R, Szczesna A, Ahn MJ, Schneider CP, Gonzalez Mella PF, Barlesi F, et al. Phase III Trial of Ipilimumab Combined With Paclitaxel and Carboplatin in Advanced Squamous Non-Small-Cell Lung Cancer. J Clin Oncol. 2017;35(30):3449-57. Epub 2017/08/31.
58. Gulley JL, Rajan A, Spigel DR, Iannotti N, Chandler J, Wong DJL, et al. Avelumab for patients with previously treated metastatic or recurrent non-small-cell lung cancer (JAVELIN Solid Tumor): dose-expansion cohort of a multicentre, open-label, phase 1b trial. Lancet Oncol. 2017;18(5):599-610. Epub 2017/04/05.
59. Hui R, Garon EB, Goldman JW, Leighl NB, Hellmann MD, Patnaik A, et al. Pembrolizumab as first-line therapy for patients with PD-L1-positive advanced non-small cell lung cancer: a phase 1 trial. Annals of oncology : official journal of the European Society for Medical Oncology / ESMO. 2017;28(4):874-81. Epub 2017/02/09.
60. Kang YK, Boku N, Satoh T, Ryu MH, Chao Y, Kato K, et al. Nivolumab in patients with advanced gastric or gastro-oesophageal junction cancer refractory to, or intolerant of, at least two previous chemotherapy regimens (ONO-4538-12, ATTRACTION-2): a randomised, double-blind, placebo-controlled, phase 3 trial. Lancet. 2017;390(10111):2461-71. Epub 2017/10/11.
61. Long GV, Atkinson V, Cebon JS, Jameson MB, Fitzharris BM, McNeil CM, et al. Standard-dose pembrolizumab in combination with reduced-dose ipilimumab for patients with advanced melanoma

(KEYNOTE-029): an open-label, phase 1b trial. Lancet Oncol. 2017;18(9):1202-10. Epub 2017/07/22.
62. Overman MJ, McDermott R, Leach JL, Lonardi S, Lenz HJ, Morse MA, et al. Nivolumab in patients with metastatic DNA mismatch repair-deficient or microsatellite instability-high colorectal cancer (CheckMate 142): an open-label, multicentre, phase 2 study. Lancet Oncol. 2017;18(9):1182-91. Epub 2017/07/25.
63. Patel MR, Ellerton J, Infante JR, Agrawal M, Gordon M, Aljumaily R, et al. Avelumab in metastatic urothelial carcinoma after platinum failure (JAVELIN Solid Tumor): pooled results from two expansion cohorts of an open-label, phase 1 trial. Lancet Oncol. 2018;19(1):51-64. Epub 2017/12/09.
64. Tang C, Welsh JW, de Groot P, Massarelli E, Chang JY, Hess KR, et al. Ipilimumab with Stereotactic Ablative Radiation Therapy: Phase I Results and Immunologic Correlates from Peripheral T Cells. Clin Cancer Res. 2017;23(6):1388-96. Epub 2016/11/01.
65. Tawbi HA, Burgess M, Bolejack V, Van Tine BA, Schuetze SM, Hu J, et al. Pembrolizumab in advanced soft-tissue sarcoma and bone sarcoma (SARC028): a multicentre, two-cohort, single-arm, open-label, phase 2 trial. Lancet Oncol. 2017;18(11):1493-501. Epub 2017/10/11.
66. Tolcher AW, Sznol M, Hu-Lieskovan S, Papadopoulos KP, Patnaik A, Rasco DW, et al. Phase Ib Study of Utomilumab (PF-05082566), a 4-1BB/CD137 Agonist, in Combination with Pembrolizumab (MK-3475) in Patients with Advanced Solid Tumors. Clin Cancer Res. 2017;23(18):5349-57. Epub 2017/06/22.
67. Weber J, Mandala M, Del Vecchio M, Gogas HJ, Arance AM, Cowey CL, et al. Adjuvant Nivolumab versus Ipilimumab in Resected Stage III or IV Melanoma. N Engl J Med. 2017;377(19):1824-35. Epub 2017/09/12.
68. Williams NL, Wuthrick EJ, Kim H, Palmer JD, Garg S, Eldredge-Hindy H, et al. Phase 1 Study of Ipilimumab Combined With Whole Brain Radiation Therapy or Radiosurgery for Melanoma Patients With Brain Metastases. Int J Radiat Oncol Biol Phys. 2017;99(1):22-30. Epub 2017/08/18.
69. Yamazaki N, Takenouchi T, Fujimoto M, Ihn H, Uchi H, Inozume T, et al. Phase 1b study of pembrolizumab (MK-3475; anti-PD-1 monoclonal antibody) in Japanese patients with advanced melanoma (KEYNOTE-041). Cancer chemotherapy and pharmacology. 2017;79(4):651-60. Epub 2017/03/12.
70. Yi JS, Ready N, Healy P, Dumbauld C, Osborne R, Berry M, et al. Immune Activation in Early-Stage Non-Small Cell Lung Cancer Patients Receiving Neoadjuvant Chemotherapy Plus Ipilimumab. Clin Cancer Res. 2017;23(24):7474-82. Epub 2017/09/28.
71. Adams S, Loi S, Toppmeyer D, Cescon DW, De Laurentiis M, Nanda R, et al. Pembrolizumab monotherapy for previously untreated, PD-L1-positive, metastatic triple-negative breast cancer: cohort B of the phase II KEYNOTE-086 study. Annals of oncology : official journal of the European Society for Medical Oncology / ESMO. 2019;30(3):405-11. Epub 2018/11/27.
72. Amaria RN, Reddy SM, Tawbi HA, Davies MA, Ross MI, Glitza IC, et al. Neoadjuvant immune checkpoint blockade in high-risk resectable melanoma. Nat Med. 2018;24(11):1649-54. Epub 2018/10/10.
73. Ariyan CE, Brady MS, Siegelbaum RH, Hu J, Bello DM, Rand J, et al. Robust Antitumor Responses Result from Local Chemotherapy and CTLA-4 Blockade. Cancer Immunol Res. 2018;6(2):189-200. Epub 2018/01/18.
74. Arkenau HT, Martin-Liberal J, Calvo E, Penel N, Krebs MG, Herbst RS, et al. Ramucirumab Plus Pembrolizumab in Patients with Previously Treated Advanced or Metastatic Biliary Tract Cancer: Nonrandomized, Open-Label, Phase I Trial (JVDF). Oncologist. 2018;23(12):1407-e136. Epub

2018/06/02.
75. Armand P, Engert A, Younes A, Fanale M, Santoro A, Zinzani PL, et al. Nivolumab for Relapsed/Refractory Classic Hodgkin Lymphoma After Failure of Autologous Hematopoietic Cell Transplantation: Extended Follow-Up of the Multicohort Single-Arm Phase II CheckMate 205 Trial. J Clin Oncol. 2018;36(14):1428-39. Epub 2018/03/28.
76. Atkins MB, Hodi FS, Thompson JA, McDermott DF, Hwu WJ, Lawrence DP, et al. Pembrolizumab Plus Pegylated Interferon alfa-2b or Ipilimumab for Advanced Melanoma or Renal Cell Carcinoma: Dose-Finding Results from the Phase Ib KEYNOTE-029 Study. Clin Cancer Res. 2018;24(8):1805-15. Epub 2018/01/24.
77. Atkins MB, Plimack ER, Puzanov I, Fishman MN, McDermott DF, Cho DC, et al. Axitinib in combination with pembrolizumab in patients with advanced renal cell cancer: a non-randomised, open-label, dose-finding, and dose-expansion phase 1b trial. Lancet Oncol. 2018;19(3):405-15. Epub 2018/02/15.
78. Bajor DL, Mick R, Riese MJ, Huang AC, Sullivan B, Richman LP, et al. Long-term outcomes of a phase I study of agonist CD40 antibody and CTLA-4 blockade in patients with metastatic melanoma. Oncoimmunology. 2018;7(10):e1468956. Epub 2018/10/06.
79. Balar AV, Castellano D, O'Donnell PH, Grivas P, Vuky J, Powles T, et al. First-line pembrolizumab in cisplatin-ineligible patients with locally advanced and unresectable or metastatic urothelial cancer (KEYNOTE-052): a multicentre, single-arm, phase 2 study. Lancet Oncol. 2017;18(11):1483-92. Epub 2017/10/03.
80. Barta SK, Zain J, MacFarlane AWt, Smith SM, Ruan J, Fung HC, et al. Phase II Study of the PD-1 Inhibitor Pembrolizumab for the Treatment of Relapsed or Refractory Mature T-cell Lymphoma. Clinical lymphoma, myeloma & leukemia. 2019. Epub 2019/04/29.
81. Boudadi K, Suzman DL, Anagnostou V, Fu W, Luber B, Wang H, et al. Ipilimumab plus nivolumab and DNA-repair defects in AR-V7-expressing metastatic prostate cancer. Oncotarget. 2018;9(47):28561-71. Epub 2018/07/10.
82. Choueiri TK, Larkin J, Oya M, Thistlethwaite F, Martignoni M, Nathan P, et al. Preliminary results for avelumab plus axitinib as first-line therapy in patients with advanced clear-cell renal-cell carcinoma (JAVELIN Renal 100): an open-label, dose-finding and dose-expansion, phase 1b trial. Lancet Oncol. 2018;19(4):451-60. Epub 2018/03/14.
83. Chung V, Kos FJ, Hardwick N, Yuan Y, Chao J, Li D, et al. Evaluation of safety and efficacy of p53MVA vaccine combined with pembrolizumab in patients with advanced solid cancers. Clinical & translational oncology : official publication of the Federation of Spanish Oncology Societies and of the National Cancer Institute of Mexico. 2019;21(3):363-72. Epub 2018/08/11.
84. D'Angelo SP, Mahoney MR, Van Tine BA, Atkins J, Milhem MM, Jahagirdar BN, et al. Nivolumab with or without ipilimumab treatment for metastatic sarcoma (Alliance A091401): two open-label, non-comparative, randomised, phase 2 trials. Lancet Oncol. 2018;19(3):416-26. Epub 2018/01/27.
85. Davar D, Wang H, Chauvin JM, Pagliano O, Fourcade JJ, Ka M, et al. Phase Ib/II Study of Pembrolizumab and Pegylated-Interferon Alfa-2b in Advanced Melanoma. J Clin Oncol. 2018:JCO1800632. Epub 2018/10/26.
86. Doi T, Piha-Paul SA, Jalal SI, Saraf S, Lunceford J, Koshiji M, et al. Safety and Antitumor Activity of the Anti-Programmed Death-1 Antibody Pembrolizumab in Patients With Advanced Esophageal Carcinoma. J Clin Oncol. 2018;36(1):61-7. Epub 2017/11/09.

87. Eggermont AMM, Blank CU, Mandala M, Long GV, Atkinson V, Dalle S, et al. Adjuvant Pembrolizumab versus Placebo in Resected Stage III Melanoma. N Engl J Med. 2018;378(19):1789-801. Epub 2018/04/17.

88. Fehrenbacher L, von Pawel J, Park K, Rittmeyer A, Gandara DR, Ponce Aix S, et al. Updated Efficacy Analysis Including Secondary Population Results for OAK: A Randomized Phase III Study of Atezolizumab versus Docetaxel in Patients with Previously Treated Advanced Non-Small Cell Lung Cancer. Journal of thoracic oncology : official publication of the International Association for the Study of Lung Cancer. 2018;13(8):1156-70. Epub 2018/05/20.

89. Gadgeel SM, Stevenson JP, Langer CJ, Gandhi L, Borghaei H, Patnaik A, et al. Pembrolizumab and platinum-based chemotherapy as first-line therapy for advanced non-small-cell lung cancer: Phase 1 cohorts from the KEYNOTE-021 study. Lung Cancer. 2018;125:273-81. Epub 2018/11/16.

90. Gandhi L, Rodriguez-Abreu D, Gadgeel S, Esteban E, Felip E, De Angelis F, et al. Pembrolizumab plus Chemotherapy in Metastatic Non-Small-Cell Lung Cancer. N Engl J Med. 2018;378(22):2078-92. Epub 2018/04/17.

91. Garassino MC, Cho BC, Kim JH, Mazieres J, Vansteenkiste J, Lena H, et al. Durvalumab as third-line or later treatment for advanced non-small-cell lung cancer (ATLANTIC): an open-label, single-arm, phase 2 study. Lancet Oncol. 2018;19(4):521-36. Epub 2018/03/17.

92. Gubens MA, Sequist LV, Stevenson JP, Powell SF, Villaruz LC, Gadgeel SM, et al. Pembrolizumab in combination with ipilimumab as second-line or later therapy for advanced non-small-cell lung cancer: KEYNOTE-021 cohorts D and H. Lung Cancer. 2019;130:59-66. Epub 2019/03/20.

93. Haag GM, Zoernig I, Hassel JC, Halama N, Dick J, Lang N, et al. Phase II trial of ipilimumab in melanoma patients with preexisting humoural immune response to NY-ESO-1. Eur J Cancer. 2018;90:122-9. Epub 2018/01/08.

94. Hodi FS, Chiarion-Sileni V, Gonzalez R, Grob JJ, Rutkowski P, Cowey CL, et al. Nivolumab plus ipilimumab or nivolumab alone versus ipilimumab alone in advanced melanoma (CheckMate 067): 4-year outcomes of a multicentre, randomised, phase 3 trial. Lancet Oncol. 2018;19(11):1480-92. Epub 2018/10/27.

95. Janjigian YY, Bendell J, Calvo E, Kim JW, Ascierto PA, Sharma P, et al. CheckMate-032 Study: Efficacy and Safety of Nivolumab and Nivolumab Plus Ipilimumab in Patients With Metastatic Esophagogastric Cancer. J Clin Oncol. 2018;36(28):2836-44. Epub 2018/08/16.

96. Lisberg A, Hunt J, Reese N, Wang T, Coluzzi P, Spiegel M, et al. A phase II study of pembrolizumab in EGFR mutant, PD-L1+, tyrosine kinase inhibitor (TKI) naïve patients with advanced NSCLC. Journal of Thoracic Oncology. 2017;12(11):S1805.

97. Liu SV, Camidge DR, Gettinger SN, Giaccone G, Heist RS, Hodi FS, et al. Long-term survival follow-up of atezolizumab in combination with platinum-based doublet chemotherapy in patients with advanced non-small-cell lung cancer. Eur J Cancer. 2018;101:114-22. Epub 2018/07/28.

98. Long GV, Atkinson V, Lo S, Sandhu S, Guminski AD, Brown MP, et al. Combination nivolumab and ipilimumab or nivolumab alone in melanoma brain metastases: a multicentre randomised phase 2 study. Lancet Oncol. 2018;19(5):672-81. Epub 2018/04/01.

99. Namikawa K, Kiyohara Y, Takenouchi T, Uhara H, Uchi H, Yoshikawa S, et al. Efficacy and safety of nivolumab in combination with ipilimumab in Japanese patients with advanced melanoma: An open-label, single-arm, multicentre phase II study. Eur J Cancer. 2018;105:114-26. Epub 2018/11/18.

100. Ott PA, Bang YJ, Piha-Paul SA, Razak ARA, Bennouna J, Soria JC, et al. T-Cell-Inflamed

Gene-Expression Profile, Programmed Death Ligand 1 Expression, and Tumor Mutational Burden Predict Efficacy in Patients Treated With Pembrolizumab Across 20 Cancers: KEYNOTE-028. J Clin Oncol. 2019;37(4):318-27. Epub 2018/12/18.
101. Paz-Ares L, Luft A, Vicente D, Tafreshi A, Gumus M, Mazieres J, et al. Pembrolizumab plus Chemotherapy for Squamous Non-Small-Cell Lung Cancer. N Engl J Med. 2018;379(21):2040-51. Epub 2018/10/04.
102. Ribas A, Medina T, Kummar S, Amin A, Kalbasi A, Drabick JJ, et al. SD-101 in Combination with Pembrolizumab in Advanced Melanoma: Results of a Phase Ib, Multicenter Study. Cancer Discov. 2018;8(10):1250-7. Epub 2018/08/30.
103. Sakamuri D, Glitza IC, Betancourt Cuellar SL, Subbiah V, Fu S, Tsimberidou AM, et al. Phase I Dose-Escalation Study of Anti-CTLA-4 Antibody Ipilimumab and Lenalidomide in Patients with Advanced Cancers. Mol Cancer Ther. 2018;17(3):671-6. Epub 2017/12/15.
104. Shitara K, Ozguroglu M, Bang YJ, Di Bartolomeo M, Mandala M, Ryu MH, et al. Pembrolizumab versus paclitaxel for previously treated, advanced gastric or gastro-oesophageal junction cancer (KEYNOTE-061): a randomised, open-label, controlled, phase 3 trial. Lancet. 2018;392(10142):123-33. Epub 2018/06/09.
105. Tarhini AA, Lee SJ, Li X, Rao UNM, Nagarajan A, Albertini MR, et al. E3611-A Randomized Phase II Study of Ipilimumab at 3 or 10 mg/kg Alone or in Combination with High-Dose Interferon-alpha2b in Advanced Melanoma. Clin Cancer Res. 2019;25(2):524-32. Epub 2018/11/14.
106. Tawbi HA, Forsyth PA, Algazi A, Hamid O, Hodi FS, Moschos SJ, et al. Combined Nivolumab and Ipilimumab in Melanoma Metastatic to the Brain. N Engl J Med. 2018;379(8):722-30. Epub 2018/08/23.
107. Le Tourneau C, Hoimes C, Zarwan C, Wong DJ, Bauer S, Claus R, et al. Avelumab in patients with previously treated metastatic adrenocortical carcinoma: phase 1b results from the JAVELIN solid tumor trial. J Immunother Cancer. 2018;6(1):111. Epub 2018/10/24.
108. Yang CJ, McSherry F, Mayne NR, Wang X, Berry MF, Tong B, et al. Surgical Outcomes After Neoadjuvant Chemotherapy and Ipilimumab for Non-Small Cell Lung Cancer. The Annals of thoracic surgery. 2018;105(3):924-9. Epub 2017/12/21.
109. Zhu AX, Finn RS, Edeline J, Cattan S, Ogasawara S, Palmer D, et al. Pembrolizumab in patients with advanced hepatocellular carcinoma previously treated with sorafenib (KEYNOTE-224): a non-randomised, open-label phase 2 trial. Lancet Oncol. 2018;19(7):940-52. Epub 2018/06/08.
110. Chung HC, Arkenau HT, Lee J, Rha SY, Oh DY, Wyrwicz L, et al. Avelumab (anti-PD-L1) as first-line switch-maintenance or second-line therapy in patients with advanced gastric or gastroesophageal junction cancer: phase 1b results from the JAVELIN Solid Tumor trial. J Immunother Cancer. 2019;7(1):30. Epub 2019/02/06.
111. Chung HC, Ros W, Delord JP, Perets R, Italiano A, Shapira-Frommer R, et al. Efficacy and Safety of Pembrolizumab in Previously Treated Advanced Cervical Cancer: Results From the Phase II KEYNOTE-158 Study. J Clin Oncol. 2019:JCO1801265. Epub 2019/04/04.
112. Disselhorst MJ, Quispel-Janssen J, Lalezari F, Monkhorst K, de Vries JF, van der Noort V, et al. Ipilimumab and nivolumab in the treatment of recurrent malignant pleural mesothelioma (INITIATE): results of a prospective, single-arm, phase 2 trial. The Lancet Respiratory medicine. 2019;7(3):260-70. Epub 2019/01/21.
113. Emens LA, Cruz C, Eder JP, Braiteh F, Chung C, Tolaney SM, et al. Long-term Clinical Outcomes and Biomarker Analyses of Atezolizumab Therapy for Patients With Metastatic

Triple-Negative Breast Cancer: A Phase 1 Study. JAMA oncology. 2019;5(1):74-82. Epub 2018/09/23.
114. Fountain E, Bassett RL, Cain S, Posada L, Gombos DS, Hwu P, et al. Adjuvant Ipilimumab in High-Risk Uveal Melanoma. Cancers. 2019;11(2). Epub 2019/02/01.
115. Katsuya Y, Horinouchi H, Seto T, Umemura S, Hosomi Y, Satouchi M, et al. Single-arm, multicentre, phase II trial of nivolumab for unresectable or recurrent thymic carcinoma: PRIMER study. Eur J Cancer. 2019;113:78-86. Epub 2019/04/17.
116. Leighl NB, Hellmann MD, Hui R, Carcereny E, Felip E, Ahn MJ, et al. Pembrolizumab in patients with advanced non-small-cell lung cancer (KEYNOTE-001): 3-year results from an open-label, phase 1 study. The Lancet Respiratory medicine. 2019;7(4):347-57. Epub 2019/03/17.
117. Loibl S, Untch M, Burchardi N, Huober J, Sinn BV, Blohmer JU, et al. A randomised phase II study investigating durvalumab in addition to an anthracycline taxane-based neoadjuvant therapy in early triple negative breast cancer - clinical results and biomarker analysis of GeparNuevo study. Annals of oncology : official journal of the European Society for Medical Oncology / ESMO. 2019. Epub 2019/05/17.
118. Makker V, Rasco D, Vogelzang NJ, Brose MS, Cohn AL, Mier J, et al. Lenvatinib plus pembrolizumab in patients with advanced endometrial cancer: an interim analysis of a multicentre, open-label, single-arm, phase 2 trial. Lancet Oncol. 2019;20(5):711-8. Epub 2019/03/30.
119. Matulonis UA, Shapira-Frommer R, Santin AD, Lisyanskaya AS, Pignata S, Vergote I, et al. Antitumor Activity and Safety of Pembrolizumab in Patients with Advanced Recurrent Ovarian Cancer: Results from the Phase 2 KEYNOTE-100 Study. Annals of oncology : official journal of the European Society for Medical Oncology / ESMO. 2019. Epub 2019/05/03.
120. Mok TSK, Wu YL, Kudaba I, Kowalski DM, Cho BC, Turna HZ, et al. Pembrolizumab versus chemotherapy for previously untreated, PD-L1-expressing, locally advanced or metastatic non-small-cell lung cancer (KEYNOTE-042): a randomised, open-label, controlled, phase 3 trial. Lancet. 2019;393(10183):1819-30. Epub 2019/04/09.
121. Nghiem P, Bhatia S, Lipson EJ, Sharfman WH, Kudchadkar RR, Brohl AS, et al. Durable Tumor Regression and Overall Survival in Patients With Advanced Merkel Cell Carcinoma Receiving Pembrolizumab as First-Line Therapy. J Clin Oncol. 2019;37(9):693-702. Epub 2019/02/07.
122. Scherpereel A, Mazieres J, Greillier L, Lantuejoul S, Do P, Bylicki O, et al. Nivolumab or nivolumab plus ipilimumab in patients with relapsed malignant pleural mesothelioma (IFCT-1501 MAPS2): a multicentre, open-label, randomised, non-comparative, phase 2 trial. Lancet Oncol. 2019;20(2):239-53. Epub 2019/01/21.
123. Squibb B-M. A Companion Study for Patients Enrolled in Prior/Parent Ipilimumab Studies. https://ClinicalTrials.gov/show/NCT00162123; 2006.
124. Squibb B-M. Study of MDX-010 in Patients With Metastatic Hormone-Refractory Prostate Cancer. https://ClinicalTrials.gov/show/NCT00323882; 2006.
125. Squibb B-M. Phase II Study for Previously Untreated Subjects With Non Small Cell Lung Cancer (NSCLC) or Small Cell Lung Cancer (SCLC). https://ClinicalTrials.gov/show/NCT00527735; 2008.
126. Squibb B-M, Medarex. Evaluation of Tumor Response to Ipilimumab in the Treatment of Melanoma With Brain Metastases. https://ClinicalTrials.gov/show/NCT00623766; 2008.
127. Squibb B-M, Medarex. Drug-Drug Interaction - 3 Arm - Carboplatin/Paclitaxel, Dacarbazine. https://ClinicalTrials.gov/show/NCT00796991; 2009.
128. Squibb B-M, Medarex. Comparison of Ipilimumab Manufactured by 2 Different Processes in Participants With Advanced Melanoma. https://ClinicalTrials.gov/show/NCT00920907; 2009.

129. Squibb B-M. Phase 3 Study of Immunotherapy to Treat Advanced Prostate Cancer. https://ClinicalTrials.gov/show/NCT01057810; 2010.
130. Institute NC. Ipilimumab With or Without Sargramostim in Treating Patients With Stage III or Stage IV Melanoma That Cannot Be Removed by Surgery. https://ClinicalTrials.gov/show/NCT01134614; 2010.
131. Squibb B-M, Inc OPU. BMS-936558 (MDX-1106) In Subjects With Advanced/Metastatic Clear-Cell Renal Cell Carcinoma (RCC). https://ClinicalTrials.gov/show/NCT01354431; 2011.
132. Institute OKC, Institute NC. Ipilimumab in Combination With Androgen Suppression Therapy in Treating Patients With Metastatic Hormone-Resistant Prostate Cancer. https://ClinicalTrials.gov/show/NCT01498978; 2012.
133. An Efficacy Study in Gastric and Gastroesophageal Junction Cancer Comparing Ipilimumab Versus Standard of Care Immediately Following First Line Chemotherapy. https://ClinicalTrials.gov/show/NCT01585987.
134. Squibb B-M. Phase II Study of Ipilimumab Monotherapy in Recurrent Platinum-sensitive Ovarian Cancer. https://ClinicalTrials.gov/show/NCT01611558; 2012.
135. Squibb B-M, Ltd OPC. Study of Nivolumab (BMS-936558) vs. Everolimus in Pre-Treated Advanced or Metastatic Clear-cell Renal Cell Carcinoma (CheckMate 025). https://ClinicalTrials.gov/show/NCT01668784; 2012.
136. Squibb B-M. Phase II Safety Study of Vemurafenib Followed by Ipilimumab in Subjects With V600 BRAF Mutated Advanced Melanoma. https://ClinicalTrials.gov/show/NCT01673854; 2012.
137. Squibb B-M. Study of BMS-936558 (Nivolumab) Compared to Docetaxel in Previously Treated Metastatic Non-squamous NSCLC. https://ClinicalTrials.gov/show/NCT01673867; 2012.
138. Institute NC. Ipilimumab With or Without High-Dose Recombinant Interferon Alfa-2b in Treating Patients With Stage III-IV Melanoma That Cannot Be Removed by Surgery. https://ClinicalTrials.gov/show/NCT01708941; 2013.
139. Squibb B-M. Study of Nivolumab (BMS-936558) Compared With Dacarbazine in Untreated, Unresectable, or Metastatic Melanoma. https://ClinicalTrials.gov/show/NCT01721772; 2013.
140. Amgen. Ipilimumab With or Without Talimogene Laherparepvec in Unresected Melanoma. https://ClinicalTrials.gov/show/NCT01740297; 2013.
141. LLC M. Randomized, Double-blind Study Comparing Tremelimumab to Placebo in Subjects With Unresectable Malignant Mesothelioma. https://ClinicalTrials.gov/show/NCT01843374; 2013.
142. Roche H-L. A Study of Atezolizumab (an Engineered Anti-Programmed Death-Ligand 1 PD-L1 Antibody) as Monotherapy or in Combination With Bevacizumab (Avastin®) Compared to Sunitinib (Sutent®) in Participants With Untreated Advanced Renal Cell Carcinoma. https://ClinicalTrials.gov/show/NCT01984242; 2014.
143. Roche H-L. A Study of Atezolizumab in Participants With Programmed Death - Ligand 1 (PD-L1) Positive Locally Advanced or Metastatic Non-Small Cell Lung Cancer. https://ClinicalTrials.gov/show/NCT02031458; 2014.
144. Squibb B-M, Ltd OPC. An Open-Label, Randomized, Phase 3 Trial of Nivolumab Versus Investigator's Choice Chemotherapy as First-Line Therapy for Stage IV or Recurrent PD-L1+ Non-Small Cell Lung Cancer (CheckMate 026). https://ClinicalTrials.gov/show/NCT02041533; 2014.
145. Squibb B-M, Ltd OPC. Trial of Nivolumab vs Therapy of Investigator's Choice in Recurrent or Metastatic Head and Neck Carcinoma (CheckMate 141). https://ClinicalTrials.gov/show/NCT02105636; 2014.

146. Squibb B-M, Ltd OPC. Nivolumab Combined With Ipilimumab Versus Sunitinib in Previously Untreated Advanced or Metastatic Renal Cell Carcinoma (CheckMate 214). https://ClinicalTrials.gov/show/NCT02231749; 2014.
147. Celgene. Safety and Efficacy Study of Nab®-Paclitaxel With CC-486 or Nab®-Paclitaxel With Durvalumab, and Nab®-Paclitaxel Monotherapy as Second/Third-line Treatment for Advanced Non-small Cell Lung Cancer. https://ClinicalTrials.gov/show/NCT02250326; 2015.
148. Sharp M, Corp. D. Study of MK-3475 (Pembrolizumab) in Recurrent or Metastatic Head and Neck Squamous Cell Carcinoma After Treatment With Platinum-based and Cetuximab Therapy (MK-3475-055/KEYNOTE-055). https://ClinicalTrials.gov/show/NCT02255097; 2014.
149. AstraZeneca, Sciences PH. Phase II Study of MEDI4736, Tremelimumab, and MEDI4736 in Combination w/ Tremelimumab Squamous Cell Carcinoma of the Head and Neck. https://ClinicalTrials.gov/show/NCT02319044; 2015.
150. AstraZeneca. A Global Study to Assess the Effects of MEDI4736 (Durvalumab), Given as Monotherapy or in Combination With Tremelimumab Determined by PD-L1 Expression Versus Standard of Care in Patients With Locally Advanced or Metastatic Non Small Cell Lung Cancer. https://ClinicalTrials.gov/show/NCT02352948; 2015.
151. Roche H-L. A Study of Atezolizumab in Combination With Carboplatin Plus (+) Nab-Paclitaxel Compared With Carboplatin+Nab-Paclitaxel in Participants With Stage IV Non-Squamous Non-Small Cell Lung Cancer (NSCLC). https://ClinicalTrials.gov/show/NCT02367781; 2015.
152. Squibb B-M, Ltd OPC. A Study of Nivolumab in Participants With Metastatic or Unresectable Bladder Cancer. https://ClinicalTrials.gov/show/NCT02387996; 2015.
153. Research ES, Development Institute I, Merck KGaA D, Germany, Serono E. Avelumab in Non-Small Cell Lung Cancer (JAVELIN Lung 200). https://ClinicalTrials.gov/show/NCT02395172; 2015.
154. Roche H-L. A Study of Atezolizumab in Combination With Bevacizumab Versus Sunitinib in Participants With Untreated Advanced Renal Cell Carcinoma (RCC). https://ClinicalTrials.gov/show/NCT02420821; 2015.
155. AstraZeneca. Study of Tremelimumab in Patients With Advanced Solid Tumors. https://ClinicalTrials.gov/show/NCT02527434; 2015.
156. Celgene. A Study of Durvalumab in Combination With Lenalidomide With and Without Dexamethasone in Adults With Newly Diagnosed Multiple Myeloma. https://ClinicalTrials.gov/show/NCT02685826; 2016.
157. Squibb B-M. A Study of Two Different Dose Combinations of Nivolumab in Combination With Ipilimumab in Subjects With Previously Untreated, Unresectable or Metastatic Melanoma. https://ClinicalTrials.gov/show/NCT02714218; 2016.
158. Corporation I, Sharp M, Corp. D. A Phase 3 Study of Pembrolizumab + Epacadostat or Placebo in Subjects With Unresectable or Metastatic Melanoma (Keynote-252 ECHO-301). https://ClinicalTrials.gov/show/NCT02752074; 2016.
159. Squibb B-M. A Safety and Efficacy Study of Multiple Administration Regimens for Nivolumab Plus Ipilimumab in Subjects With Melanoma. https://ClinicalTrials.gov/show/NCT02905266; 2016.
160. Squibb B-M. An Investigational Immuno-Therapy Safety and Efficacy Study of Multiple Administration Regimens for Nivolumab Plus Ipilimumab in Subjects With Renal Cell Carcinoma. https://ClinicalTrials.gov/show/NCT03029780; 2017.
